# Arboviruses and the Challenge to Establish Systemic and Persistent Infections in Competent Mosquito Vectors: The Interaction With the RNAi Mechanism

**DOI:** 10.3389/fphys.2019.00890

**Published:** 2019-07-11

**Authors:** Jisheng Liu, Luc Swevers, Anna Kolliopoulou, Guy Smagghe

**Affiliations:** ^1^School of Life Sciences, Guangzhou University, Guangzhou, China; ^2^Institute of Biosciences and Applications, National Centre of Scientific Research “Demokritos”, Athens, Greece; ^3^Department of Plants and Crops, Faculty of Bioscience Engineering, Ghent University, Ghent, Belgium

**Keywords:** arbovirus, mosquito, persistent infection, RNAi, siRNA, piRNA, antiviral defense, viral suppressor of RNAi

## Abstract

Arboviruses are capable to establish long-term persistent infections in mosquitoes that do not affect significantly the physiology of the insect vectors. Arbovirus infections are controlled by the RNAi machinery via the production of viral siRNAs and the formation of RISC complexes targeting viral genomes and mRNAs. Engineered arboviruses that contain cellular gene sequences can therefore be transformed to “viral silencing vectors” for studies of gene function in reverse genetics approaches. More specifically, “ideal” viral silencing vectors must be competent to induce robust RNAi effects while other interactions with the host immune system should be kept at a minimum to reduce non-specific effects. Because of their inconspicuous nature, arboviruses may approach the “ideal” viral silencing vectors in insects and it is therefore worthwhile to study the mechanisms by which the interactions with the RNAi machinery occur. In this review, an analysis is presented of the antiviral RNAi response in mosquito vectors with respect to the major types of arboviruses (alphaviruses, flaviviruses, bunyaviruses, and others). With respect to antiviral defense, the exo-RNAi pathway constitutes the major mechanism while the contribution of both miRNAs and viral piRNAs remains a contentious issue. However, additional mechanisms exist in mosquitoes that are capable to enhance or restrict the efficiency of viral silencing vectors such as the amplification of RNAi effects by DNA forms, the existence of incorporated viral elements in the genome and the induction of a non-specific systemic response by Dicer-2. Of significance is the observation that no major “viral suppressors of RNAi” (VSRs) seem to be encoded by arboviral genomes, indicating that relatively tight control of the activity of the RNA-dependent RNA polymerase (RdRp) may be sufficient to maintain the persistent character of arbovirus infections. Major strategies for improvement of viral silencing vectors therefore are proposed to involve engineering of VSRs and modifying of the properties of the RdRp. Because of safety issues (pathogen status), however, arbovirus-based silencing vectors are not well suited for practical applications, such as RNAi-based mosquito control. In that case, related mosquito-specific viruses that also establish persistent infections and may cause similar RNAi responses may represent a valuable alternative solution.

## Introduction

RNA interference (RNAi) has become an important tool to analyze gene function in eukaryotes, including insects. RNAi technology is based on the administration of dsRNA that will trigger the degradation of homologous cellular mRNAs. Because of the specificity of gene silencing effects, RNAi has become a powerful reverse genetics tool for analysis of gene function, especially in non-model organisms, which include many insects (Bellés, 2010; Huvenne and Smagghe, 2010; Santos et al., 2014; Schmitt-Engel et al., 2015; Whitten and Dyson, 2017; Lopez et al., 2019). However, in many insects the process of gene silencing following injection or feeding of dsRNA is not very robust which has stimulated research to develop new methods of delivery of dsRNA to increase efficiency (Zhang et al., 2013; Joga et al., 2016). One delivery system that is proposed is based on the use of recombinant viruses that naturally trigger the RNAi response in insects (Kolliopoulou et al., 2017). This strategy, termed virus-induced gene silencing (VIGS), was first pioneered in plants and employs the efficiency by which viruses can enter and replicate in cells. However, a disadvantage of the use of viruses is that robust replication can cause cellular damage and induce the immune response that will obscure the interpretation of the phenotypes that correspond to the host sequences that are integrated in the recombinant viral silencing vectors. It is therefore proposed that viral silencing vectors should be engineered carefully such that a moderate level of replication is achieved, capable to deliver sufficient dsRNA molecules to activate the RNAi machinery, while simultaneously avoiding to interfere with cellular function or to induce the immune response. For this reason, the development of this technique can benefit and learn from the study of arbovirus infections in insect vectors because they have been reported to both interact with the RNAi machinery and to be entirely non-pathogenic (Myles et al., 2008; Blair, 2011; Rückert et al., 2014; Goic et al., 2016). In insect vectors, arboviruses are presumed to strike a delicate balance between replication kinetics and avoidance of the immune response (O’Neal et al., 2014; Samuel et al., 2018). As a complicating factor, avoidance of the immune response regularly includes suppression of RNAi, which makes the engineering of viruses as both efficient and specific silencing vectors particularly challenging.

Arbovirus infections of mosquito vectors represent an interesting system of how persistent and systemic virus infections become established and maintained in insect hosts. As such, studies of arbovirus infections can provide important insights to inspire the optimization of viral silencing vectors in other insects. As a background, this review will investigate the process of RNAi as an antiviral response during arbovirus infections and to what extent arboviruses (in their persistent state) can function as RNAi transduction vectors in mosquitoes. While RNAi is considered the most important factor that modulates arbovirus–mosquito interactions, it should be noted that other pathways and processes (e.g., innate immune response pathways, stress response, apoptosis and autophagy, alternative RNA degradation pathways, microbiome; Kingsolver et al., 2013; Swevers et al., 2018) may also have a significant impact. In this review, focus will be on the RNAi response in which the small interfering RNA (siRNA) pathway (“exo-RNAi”; see section “The RNAi Machinery in Mosquito Vectors”) is considered the major defense mechanism together with possible minor contributions from the PIWI-associated RNA (piRNA) and microRNA (miRNA) mechanisms.

## The Nature of Arbovirus Infections in Mosquito Vectors

Arboviruses or arthropod-borne viruses refer to a non-taxonomic group of viruses that are transmitted to humans and livestock by arthropod vectors, typically insects (mosquitoes, sandflies, black flies, biting midges; all Diptera) and ticks (Acari, Arachnida). In this review, most attention is focused on mosquitoes because of the wealth of information that is available, while other insect or tick vectors are occasionally also mentioned if relevant information is available for discussion.

Most arboviruses are RNA viruses and characterized by monopartite linear (+) ssRNA genome [*Togaviridae* (genus Alphavirus), *Flaviviridae*], monopartite linear (-) ssRNA genome (*Rhabdoviridae*), segmented linear (-) or ambisense ssRNA genome (Bunyavirales) and segmented linear dsRNA genome (*Reoviridae*). While arboviruses can cause some of the most devastating diseases in humans (hemorrhagic disease and encephalitis-like illnesses), a distinguishing feature of arbovirus infection in mosquitoes is the establishment of a non-pathogenic, persistent state (Lambrechts and Scott, 2009). During the acute phase of infection, viral replication initially can reach high levels but viral titers subsequently become modulated to low levels during the phase of persistence (Myles et al., 2008; Fragkoudis et al., 2008). For establishment of the persistent state, a delicate balance between the virus and the immune system of the mosquito is necessary to regulate replication and maintain viral presence without causing significant adverse effects that could affect the transmission efficiency to a new host. To optimize transmission, it is indeed essential that arbovirus infections do not result in a decrease of mosquito survival because mortality before completing the transmission cycle is predicted to have a large impact on vectorial capacity (Black and Moore, 2005).

Mosquito vector competence is defined as the overall capacity of the insect vector to become orally infected by the virus after a blood meal and to transmit the virus to the next vertebrate host (Smith et al., 2012). Arboviruses therefore must be capable to infect and replicate in the midgut epithelial cells of the mosquito vector, to escape from the midgut cells to disseminate in the hemolymph and secondary insect tissues (where secondary viral replication occurs), and finally to infect the salivary glands from which progeny virus in the saliva is transmitted to the vertebrate host during a blood meal (Hardy et al., 1983; Franz et al., 2015). Depending on the particular virus-mosquito vector pair, dissemination of virus from the midgut to other tissues typically is observed between 3 and 7 days post-infection (p.i.), while mosquitoes become competent for virus transmission through the saliva between 5 and 14 days p.i. (Ebel et al., 2005; Carissimo et al., 2015; Rückert et al., 2017). Virus can persist for periods up to 4 weeks in midgut and salivary glands of infected mosquitoes (Girard et al., 2005). The “extrinsic incubation period,” defined as the interval between acquisition and transmission, is thought to be modulated extensively by the antiviral immune response, which includes RNAi, in the mosquito vector (Cheng et al., 2016).

## The RNAi Machinery in Mosquito Vectors

**FIGURE 1:**
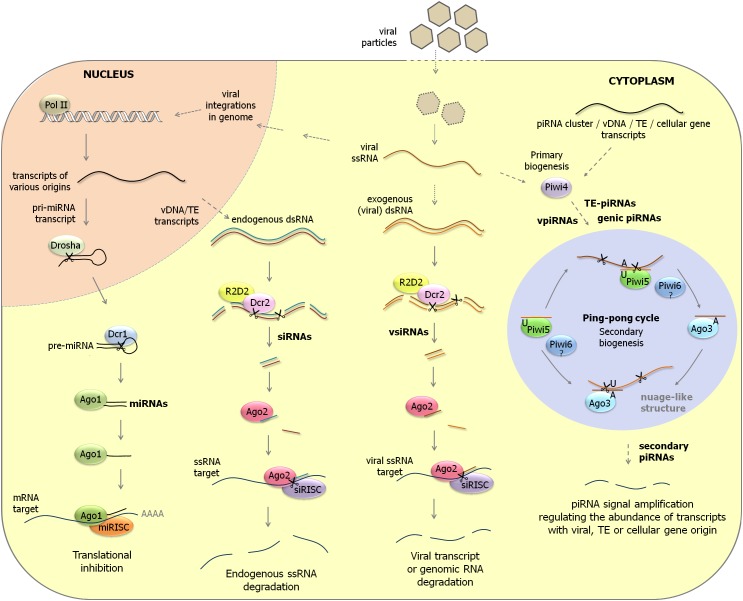
Overview of RNAi pathways in aedine mosquitoes. **(Left)** miRNA pathway. Primary miRNA transcripts are processed by Drosha in the nucleus to pre-miRNAs. After their transport to the cytoplasm, pre-miRNAs undergo further cleavage by Dicer-1 to mature miRNAs. miRISC complexes containing Ago-1 regulate cellular gene expression by translation inhibition after hybridization to mRNA targets. **(Middle)** siRNA pathway. Long dsRNA precursors that have endogenous (transposable elements, viral DNA forms) or exogenous (viral replication intermediates) origin are cleaved by Dicer-2 and its co-factor R2D2 to siRNAs and vsiRNAs. siRISC complexes containing Ago-2 subsequently scan parasitic RNA populations (transposon transcripts, viral transcripts and genomic RNAs, RNAs derived from viral integrations in genome) to trigger their destruction. **(Right)** piRNA pathway. In aedine mosquitoes, an expansion of PIWI-class Argonaute genes is observed which are expressed in somatic tissues and are involved in transposon control but possibly also in antiviral defense and cellular gene regulation. ssRNA precursors from various origins (transposable elements, viral mRNAs and genomic RNAs, transcripts from viral DNA forms, cellular gene transcripts) are processed to primary piRNAs by a Dicer-independent mechanism. While Piwi-4 does not directly interact with piRNAs, it was proposed that it acts as an important factor to activate the production of secondary piRNAs by the ping-pong mechanism. In the ping-pong cycle, piRNAs of antisense orientation (U1 bias) are mostly associated with Piwi-5 and possibly also with Piwi-6. On the other hand, piRNAs of sense orientation (A10 logo) are loaded by Ago-3. The piRNA ping-pong cycle is considered an important amplification mechanism to regulate the abundance of transcripts of transposon, viral or cellular origin. pri-miRNA, primary miRNA; pre-miRNA, precursor miRNA; vDNA, viral DNA form; TE, transposable element.

Insects are characterized by three RNAi pathways (Campbell et al., 2008; Blair, 2011; Donald et al., 2012; Rückert et al., 2014; O’Neal et al., 2014; Dowling et al., 2016). An overview of the three RNAi pathways that is focused on aedine mosquitoes is presented in Figure 1 and discussed below.

MicroRNAs (miRNAs) originate from nuclear genes and regulate cellular gene expression at the posttranscriptional level. The miRNA machinery is conserved among insects and consists of Drosha and Pasha, that process the primary miRNA transcripts in the nucleus; Dicer-1 (Dcr-1) and Loquacious (Loqs), that carry out further processing to generate ∼22 nt mature miRNAs in the cytoplasm; and Argonaute-1 (Ago-1) that constitutes the central factor in the miRNA-induced silencing complex (miRISC).

The main function of the siRNA pathway is the defense against invading nucleic acids such as viruses (called “exo-RNAi” because of the exogenous origin of the dsRNA trigger) and transposable elements in the genome (“endo-RNAi”). In this pathway, long dsRNAs are processed by Dicer-2 (Dcr-2) and its co-factor R2D2 (or a specific isoform of Loqs in the case of the defense against transposons) to ∼21 nt siRNAs which are subsequently loaded into siRISC containing Argonaute-2 (Ago-2) to silence viral genes and transposons. Factors in the siRNA pathway have undergone accelerated evolution as a consequence of constant adaptations during the host–virus arms race (Obbard et al., 2006). Mosquito species that are commonly used in research, such as *Aedes aegypti* (yellow fever mosquito), *Aedes albopictus* (Asian tiger mosquito) and *Anopheles gambiae* (African malaria mosquito), are characterized by single genes each of *dcr-2* and *ago-2*, while a duplication of *ago-2* has occurred in the culicine mosquito *Culex pipiens* (Campbell et al., 2008; Lewis et al., 2016).

The third pathway, PIWI-interacting RNA (piRNA) pathway, was initially identified as a defense mechanism against transposition of mobile elements in the germline of *Drosophila* (Handler et al., 2013). Biogenesis of 24–29 nt piRNAs occurs in a Dicer-independent manner and the effector RISC complexes in the piRNA pathway employ members of the PIWI subclass of Argonaute proteins [Piwi, Aubergine (Aub) and Ago-3 in *Drosophila*] (Miesen et al., 2016b). The piRNA pathway in *Drosophila* consists of two branches: (1) the primary (intermediate) pathway in which (single-stranded) transcripts from discrete genomic loci termed piRNA clusters (consisting of remnants of transposable elements) are processed by the nuclease Zucchini, followed by loading of piRNAs in Piwi-containing effector complexes to perform transcriptional gene silencing in the nucleus; and (2) the secondary (mature) pathway that occurs in the cytoplasm and is characterized by the ping-pong amplification loop. In the ping-pong mechanism, transposon (sense) transcripts are cleaved by Aub (loaded with antisense piRNAs) to generate complementary piRNAs (sense orientation) that are loaded in Ago-3-complexes. Because sense piRNA-Ago-3 complexes will subsequently target again transposon sequences in the antisense orientation, an amplification loop is created of production of both mature antisense and sense piRNAs that expedite transposon silencing. In the ping-pong mechanism, the 5′-end of the guide piRNA is located at exactly 10 nt from the 5′-end of the cleavage site. Because the 5′-ends of piRNAs associated with Piwi or Aub have a characteristic U-bias, products are generated with A at the 10th position, that become regularly associated with Ago-3. The U1/A10 bias and the 10 nt overlap between the 5′-ends in complementary small RNAs are therefore regarded as hallmarks of the ping-pong mechanism in the piRNA pathway (Senti and Brennecke, 2010).

However, while the piRNA pathway is mainly restricted to the germline in *Drosophila*, research in insects and other arthropods revealed an ancestral role for piRNAs in defense against transposable elements in both somatic and germline tissues (Lewis et al., 2018). Furthermore, while *ago-3* is conserved as a single gene in many insects, the lineage of *piwi* and *aub* is characterized by a high rate of duplication events in dipteran insects that reflects the evolutionary arms race against transposable elements (Lewis et al., 2016). More specifically, the existence of *piwi* and *aub* genes (that have separate functions in transcriptional and post-transcriptional silencing, respectively) in *Drosophila* reflects the duplication of an ancestral gene (“*piwi/aub*”) at the base of the Brachycera. In aedine and culicine mosquitoes, on the other hand, the hypothetical “*piwi/aub*” ancestral gene has undergone a remarkable expansion (seven genes in *Ae. aegypti*, nine genes in *Ae. albopictus*, and six genes in *Cx. pipiens quinquefasciatus*) (Campbell et al., 2008; Lewis et al., 2016; Wang et al., 2018). By contrast, the genome of *An. gambiae* encodes one *ago-3* and two Piwi-related genes, named *ago-4* and *ago-5*, that are orthologs of *piwi/aub* (Campbell et al., 2008; Vodovar et al., 2012; Macias et al., 2014). Also the genome of the midge, *Culicoides sonorensis* (Ceratopogonidae) encodes one *ago-3* and two “*aub/piwi*” genes (Lewis et al., 2016).

More specific data with respect to expression of PIWI subclass genes in tissues of mosquitoes are available for *Ae. aegypti* and *Ae. albopictus*. Of the seven Piwi-related genes and one *ago-3* gene that are identified in the genome of *Ae. aegypti*, *piwi-4*, *piwi-5*, *piwi-6*, and *ago-3* are clearly expressed in the somatic tissues of the mosquito (Morazzani et al., 2012; Miesen et al., 2015). On the other hand, *piwi-1-3* expression seems to be germline specific while *piwi-7* is only present in the early embryo (Akbari et al., 2013). A recent study shows increased expression of Piwi-related and *ago-3* genes in ovarian tissue compared to midgut in *Cx. quinquefasciatus* mosquitoes (Rückert et al., 2019).

The expansion of PIWI subclass genes in the genome of *Ae. albopictus* consists of two *ago-3* homologs, six homologs of *ago-4* of *An. gambiae* (*piwi-1-6*, related to *Ae. aegypti* *piwi-1-*4) and three homologs of *ago-5* of *An. gambiae* (*piwi-7-9*, related to *Ae. aegypti piwi-5-7*) (Wang et al., 2018). In *Ae. albopictus* adult females, mRNAs of the two *ago-3* paralogs and *piwi-1-7* can be readily detected while *piwi-8* and *-9* are highly expressed in embryos. In midgut tissue, only mRNAs of *piwi-5-7* and the two *ago-3* paralogs are present.

## Evidence of Interactions of Arboviruses With the RNAi Machinery

Even before the process of RNAi was clarified in animals (Fire et al., 1998), it was observed that double subgenomic recombinant Sindbis viruses (SINV; Alphavirus) that express sequences of genetically unrelated RNA viruses could induce resistance against infection of these viruses in mosquito cells (Olson et al., 2002). This strategy, called “pathogen-derived resistance” or “RNA-mediated cross-protection between viruses,” could provide protection against infection of dengue virus (DENV; *Flaviviridae*) (Gaines et al., 1996; Olson et al., 1996; Adelman et al., 2001) as well as La Crosse virus (LACV; *Bunyavirales*) (Powers et al., 1996). Pathogen-derived resistance was sequence-specific, since it was effective against one specific DENV serotype while other serotypes were not affected, and it was mediated at the level of RNA since the use of antisense constructs or the introduction of artificial stop codons in sense constructs did not affect efficiency. The process is now understood to be triggered by RNAi in which viral replication intermediates with dsRNA structure are recognized by Dicer to generate siRNAs that can target the genetically unrelated viruses (in addition to targeting the SINV viruses) (Bronkhorst and van Rij, 2014; Gammon and Mello, 2015). This phenomenon was observed in both mosquitoes (*Ae. aegypti* and *Ae. triseriatus*) as well as in C6/36 cells that are derived from *Ae. albopictus* (Gaines et al., 1996; Powers et al., 1996; Olson et al., 2002).

The sensitivity of arbovirus infections to silencing by the RNAi mechanism was also demonstrated in transformed C6/36 cell lines that express an inverted repeat RNA corresponding to the prM gene of DENV-2 (Adelman et al., 2002). Eight out of 18 transformed C6/36 cell lines that expressed the inverted repeat RNA were resistant to DENV-2 challenge which was correlated with the production of small RNAs from the hairpin construct. This strategy was subsequently further extended to generate transgenic *Ae. aegypti* mosquitoes that expressed an RNA hairpin targeting the prM region of DENV-2 and that were shown to be refractory to DENV-2 infection (Franz et al., 2006, 2014). Induction of RNA hairpin expression by the midgut-specific *carboxypeptidase A* promoter occurred after feeding of a blood meal and was shown to provide protection against DENV-2 infection by oral feeding. The involvement of the RNAi mechanism was confirmed following the detection of mainly 21 nt siRNAs originating from the hairpin and by the loss of protection against DENV-2 infection following knock-down of *ago-2*. Protection was strong against DENV-2 infection but not against other DENV serotypes or CHIKV (Alphavirus), confirming the specificity of the antiviral RNAi response. Introgression of the RNA hairpin transgene into mosquitoes of another genetic background resulted in a change from the susceptible to the refractory phenotype (Franz et al., 2014). A homozygous *Ae. aegypti* mosquito line that is refractory to DENV-2 infection with minimal fitness loss was selected that can be used for studies of spread within mosquito populations. Furthermore, a complementary study that used transgenic mosquitoes that express an RNA hairpin targeting prM of DENV-2 in the female salivary glands likewise resulted in inhibition of viral infection of the salivary glands as well as in a significant diminution of the transmission of DENV-2 (Mathur et al., 2010).

## Antiviral Defense by the exo-RNAi Pathway in Mosquitoes

Interactions of viral infections with the RNAi machinery in *Drosophila* are usually assessed by three criteria: (1) the production of viral siRNAs (vsiRNAs); (2) increase in viral replication and mortality in *ago-2* and *dcr-2* mutants; (3) the presence of genes in the viral genome that encode VSRs (Bronkhorst and van Rij, 2014; Pijlman, 2014). When these criteria are applied to mosquitoes, two difficulties are encountered: (1) a *dcr-2* mutant mosquito line only recently has become available for *Ae. aegypti* (Samuel et al., 2016; Varjak et al., 2017b) and therefore the assessment of the involvement of *ago-2* and *dcr-2* in earlier studies and in other mosquitoes was carried out by RNAi-mediated knock-down, which is considered an inefficient process; and (2) arboviruses in general are considered to be lacking VSR genes; although a few convincing candidates were proposed, conclusive evidence within the native viral context or in the natural vector was not achieved (O’Neal et al., 2014; Samuel et al., 2018). Although the evidence is less robust as in *Drosophila*, the involvement of the exo-RNAi pathway in the defense against arboviral infections in mosquitoes nevertheless does not seem to be in doubt. However, research has also indicated that the efficiency of RNA-mediated silencing can be modulated by environmental factors, such as temperature, with repercussions on the antiviral response, e.g., the higher arbovirus infection levels observed at cooler temperature (Adelman et al., 2013).

When viral small RNAs (vsiRNAs but also viral piRNAs; see section “What is the Role of the piRNA Pathway in Antiviral Defense?”), obtained after deep sequencing analysis, are mapped to the viral genomes, cold and hot spot regions of under- and over-representation, respectively, are typically observed. The occurrence of these regions may result from preferential processing by Dcr-2 but other causes, such as differential stability of small RNAs and preferential targeting of regions for production of viral DNA forms (considered as an amplification mechanism for viral small RNA production; see section “Amplification of the Antiviral RNAi Response and Potential Establishment of Immune Memory”) need to be considered. Moreover, the preferential detection may be the result of technical issues that occur in deep sequencing experiments, such as library preparation (Linsen et al., 2009). Ideally, the abundance of viral small RNAs corresponding to specific regions of the viral genome should be confirmed by other biochemical or molecular methods (e.g., Northern blot, qPCR).

Below follows a detailed analysis of the function of the exo-RNAi pathway as an antiviral defense mechanism in mosquitoes. Production of vsiRNAs is summarized in Table 1. An overview of the documented involvement of factors in the exo-RNAi pathway is presented in Table 2.

**Table 1:** Viral small RNAs produced during arbovirus infections of mosquitoes.

Virus	Mosquito	Tissue	Viral small RNAs	References
***Alphavirus***				
CHIKV	*Ae. aegypti*	Whole body	21 nt vsiRNA	Morazzani et al., 2012
			26–29 nt vpiRNA	
CHIKV	*Ae. albopictus*	Whole body	21 nt vsiRNA	Morazzani et al., 2012
		Head-thorax	26–29 nt vpiRNA	
CHIKV	*Ae. albopictus*	Whole body	21 nt vsiRNA	Goic et al., 2016
			27–29 nt vpiRNA	
ONNV	*An. gambiae*	Whole body	21 nt vsiRNA	Myles et al., 2009
ONNV	*An. gambiae*	Whole body	21 nt vsiRNA	Carissimo et al., 2015
SINV	*Ae. aegypti*	Whole body	21 nt vsiRNA	Myles et al., 2008
SINV	*Cx. pipiens*	Whole body	21 nt vsiRNA	Miesen et al., 2016b
***Flavivirus***				
DENV	*Ae. aegypti*	Whole body	21 nt vsiRNA	Scott et al., 2010
DENV	*Ae. aegypti*	Whole body	<20 nt usRNA	Hess et al., 2011
			20–23 nt vsiRNA	
			24–30 nt vpiRNA	
DENV	*Ae. albopictus*	Whole body	13–19 nt usRNA	Wang et al., 2018
		Midgut	20–23 nt vsiRNA	
			24–30 nt vpiRNA	
USUV	*Cx. pipiens*	Whole body	21 nt vsiRNA	Fros et al., 2015
WNV	*Ae. aegypti*	Midgut	21 nt vsiRNA	Rückert et al., 2019
WNV	*Cx. pipiens quinquefaciatus*	Midgut	20–22 nt vsiRNA	Brackney et al., 2009
WNV	*Cx. pipiens*	Whole body	21 nt vsiRNA	Fros et al., 2015
WNV	*Cx. pipiens*	Whole body	21 nt vsiRNA	Göertz et al., 2016
WNV	*Cx. quinquefasciatus*	Midgut,	21 nt vsiRNA	Rückert et al., 2019
		salivary glands		
**Bunyaviridae**				
RVFV	*Ae. aegypti*	Whole body	21 nt vsiRNA	Dietrich et al., 2017a
			25–30 vpiRNA	
RVFV	*Ae. vexans*	Whole body	21 nt vsiRNA	Dietrich et al., 2017a
			27–30 vpiRNA	
RVFV	*Cx. quinquefasciatus*	Whole body	19 nt usRNA	Dietrich et al., 2017a
			21 nt vsiRNA	
			25–29 vpiRNA	


**Table 2:** RNAi factors that affect arbovirus replication in mosquitoes.

Virus	Mosquito	RNAi factor	Phenotype	References
***Alphavirus***				
CHIKV	*Ae. aegypti*	Ago-2 (RNAi knockdown)	Increase in viral titer (midgut and head)	McFarlane et al., 2014
ONNV	*An. gambiae*	Ago-2, Ago-3 (no effect for Ago-1, Ago-4, Ago-5) (RNAi knockdown)	Increase in viral titers	Keene et al., 2004
ONNV	*An. gambiae*	Ago-2 (RNAi knockdown)	Increase in viral titer (infection by injection)	Waldock et al., 2012
ONNV	*An. gambiae*	Ago-2 (RNAi knockdown)	Increase in systemic infection (infection by feeding)	Carissimo et al., 2015
SINV	*Ae. aegypti*	Ago-2, Dcr-2, TSN (RNAi knockdown)	Increased viral replication (mortality not affected)	Campbell et al., 2008
SINV	*Ae. aegypti*	*dcr-2* mutant	Increased viral replication Increased mortality	Samuel et al., 2016
SINV	*Ae. aegypti*	Dcr-2 (induction of RNA hairpin in midgut upon feeding)	Increased viral titers Increased infection of midgut Increased dissemination of virus to other tissues	Khoo et al., 2010
***Flavivirus***				
DENV	*Ae. aegypti*	Dcr-2, R2D2, Ago-2 (RNAi knockdown)	Increase in viral titer Increase in viral transmission	Sánchez-Vargas et al., 2009
YFV	*Ae. aegypti*	*dcr-2* mutant	Increase in viral replication	Samuel et al., 2016


### Alphaviruses

The most studied arboviruses of the Alphavirus genus (family: *Togaviridae*) are Sindbis virus (SINV), Semliki Forest virus (SFV), Chikungunya virus (CHIKV) and o’nyong-nyong virus (ONNV). Alphaviruses are characterized by a single linear (+) ssRNA genome (11–12 kb) that also serves as the mRNA for the non-structural polyprotein while a subgenomic RNA produced during infection functions as the source for the structural proteins. Under natural conditions, SINV circulates between *Culex* mosquitoes and birds, while humans act as “dead-end” hosts. Both SFV and CHIKV are transmitted to humans by *Aedes* species while the primary vectors of ONNV are anopheline mosquitoes (Atkins, 2013).

#### SINV

Although SINV is not naturally transmitted by *A. aegypti*, particular genotypes can establish persistent midgut infections after an infectious blood meal and spread to other tissues at high frequency (Campbell et al., 2008). RNAi-mediated knock-down of *ago-2*, *dcr-2* and to a lesser extent, the RISC component *Tudor staphylococcal nuclease* (*TSN*), resulted in enhancement of viral infection by feeding but did not affect mortality. Increased replication of recombinant SINV vectors was also observed in *dcr-2* null mutant *Ae. aegypti* mosquitoes (and was associated with increased mortality; Samuel et al., 2016). Injection of dsRNA targeting the viral non-structural protein nsP3, on the other hand, provided strong protection against viral infection. Interestingly, different strains of recombinant SINV exhibited differences in the rate of viral replication which could be negatively correlated with the production of viral small RNAs (Campbell et al., 2008). Components of the RNAi machinery (Ago-2, Dcr-2, TSN) showed a transcriptional response after ingestion of a blood meal and after oral viral infection, while also changes in Ago-2 protein levels were observed. Another study observed down-regulation of *dcr-2* in SINV-infected *Ae. aegypti* mosquitoes during late infection (Sanders et al., 2005).

Injection of SINV in *Ae. aegypti* mosquitoes resulted in the production of vsiRNAs predominantly of 21 nt size (Myles et al., 2008). VsiRNAs could be mapped along the length of the viral genome but showed regions of preferential accumulation (hot spots, which are typically observed in all virus infections). A significant bias for the positive strand was also observed, indicating that vsiRNAs derive from both replication intermediates and structured regions in viral RNA transcripts (Campbell et al., 2008; Myles et al., 2008, 2009).

Studies of oral infections of SINV in *Ae. aegypti* mosquitoes established that the RNAi response in midgut tissue was important to prevent dissemination of the virus to other tissues of the body (Khoo et al., 2010, 2013). Silencing of expression of *dcr-2* specifically in midgut tissue of *Ae. aegypti* mosquitoes immediately following an infectious blood meal of recombinant SINV indeed resulted in increased viral titers and increased infection rate of the midgut as well as increased dissemination to other tissues (Khoo et al., 2010). Similar results were obtained in transgenic *Ae. aegypti* that constitutively express the B2 RNAi inhibitor of Flock house virus (FHV; *Nodaviridae*; see also section “Evidence for Presence of VSR Genes in Arboviral Genomes” for a discussion of RNAi inhibitors) although in this case only increased dissemination of SINV from midgut to other tissues was observed (Khoo et al., 2013).

#### ONNV

Anopheline mosquitoes are mainly known for the transmission of malaria parasites while their vectoring of arboviruses seems limited. One exception is ONNV that is transmitted by *An. funestus* and *An. gambiae* mosquitoes (Rezza et al., 2017). When recombinant ONNV with a GFP reporter cassette was injected in *An. gambiae* mosquitoes, its replication and dissemination could be inhibited by dsRNA targeting the non-structural protein nsP3 (Keene et al., 2004). On the other hand, silencing of *ago-2* (siRNA pathway) and *ago-3* (piRNA pathway; see also section “Analysis of the Role of the piRNA Pathway in Antiviral Defense in Mosquito Cell Lines”) but not *ago-1* (miRNA pathway) or the Piwi/Aub-related *ago-4* and *ago-5* (piRNA pathway) (Lewis et al., 2016) resulted in an increase in viral titers (Keene et al., 2004).

Injection of ONNV in *A. gambiae* mosquitoes resulted in the production of 21 nt vsiRNAs that show similar properties to that observed following injection of SINV in *Ae. aegypti* mosquitoes (discussed above; Campbell et al., 2008; Myles et al., 2008, 2009).

However, in contrast to what is observed during midgut infection of *Ae. aegypti* mosquitoes with SINV, a more recent study that investigated in more detail the early stages of infection of *An. gambiae* with ONNV revealed that the RNAi response was not important to control the primary infection of midgut cells with an infectious blood meal [in contrast to the immune deficiency (Imd) and Janus kinase/signal transducer and activator of transcription (JAK/STAT) pathways; Carissimo et al., 2015]. The data also point to complementary immune responses in midgut and systemic compartments since the RNAi response was antiviral during infections after injection of ONNV, in contrast to the JAK-STAT and Imd pathways (Waldock et al., 2012). While vsiRNAs are produced during primary infection of the midgut by ONNV (Carissimo et al., 2015), they do not seem to provide protection at the early stage in the midgut and may function as a signal (rather than an effector) to influence responses in the systemic compartment during later infection.

#### CHIKV

When *Ae. aegypti* and *Ae. albopictus* mosquitoes were injected with CHIKV, vsiRNAs (21 nt) were produced but also viral small RNAs of piRNA size (23–30 nt) (Morazzani et al., 2012; Goic et al., 2016) (see also section “What is the Role of the piRNA Pathway in Antiviral Defense?” for discussion of the possible role of piRNAs in antiviral defense). However, silencing experiments of genes of the RNAi pathway to determine their effect on CHIKV replication in *Ae. aegypti* mosquitoes have only been carried out for *ago-2* for which an antiviral effect was revealed in both midgut and head tissue (McFarlane et al., 2014).

### Flaviviruses

The most studied viruses of the Flavivirus genus (family: *Flaviviridae*) are DENV (present as four serotypes), West Nile virus (WNV) and Zika virus (ZIKV). Flaviviruses are characterized by a single linear (+) ssRNA genome (10–11 kb) that also functions as mRNA and encodes a single polyprotein that is processed to both structural and non-structural proteins by viral and host proteases. Both DENV and ZIKV (together with the alphavirus CHIKV) are transmitted to humans by *Ae. aegypti* and *Ae. albopictus* mosquitoes. The primary vectors for transmission of WNV are *Culex* mosquitoes with birds as preferential and humans as “dead-end” hosts, respectively (Huang et al., 2014).

#### DENV

RNAi modulates DENV replication at different infection stages since knock-down of the siRNA machinery factors *dcr-2*, *r2d2* and *ago-2* in *Ae. aegypti* mosquitoes prior to an infectious blood meal affects infection prevalence, dissemination of the virus from the midgut to the salivary glands, viral titer and viral transmission via the saliva (Sánchez-Vargas et al., 2009). Increased expression of *dcr-2* and *ago-2* mRNA was observed during early infection of *Ae. aegypti* mosquitoes with DENV-2 while during later stages also *TSN* mRNA was induced (Hess et al., 2011).

Small RNA sequencing revealed vsiRNAs of 21 nt size following oral infection of *Ae. aegypti* mosquitoes with DENV-2 (Scott et al., 2010; Hess et al., 2011). Nearly equal ratios of positive and negative sense vsiRNA reads were detected suggesting that dsRNA replication intermediates constitute a major source for processing by Dcr-2. However, compared to infections with alphaviruses, the proportion of reads corresponding to vsiRNAs was very low in the case of DENV, possibly indicating low viral replication or effective sequestration of dsRNA triggers in cellular membrane vesicles during replication/transcription (Scott et al., 2010). During oral infection of *Ae. albopictus* mosquitoes with DENV-2, vsiRNAs (20–23 nt) were detected predominantly in whole body samples, in contrast to other classes of viral small RNAs that were more prevalent in midgut (Wang et al., 2018; see also section “Production of VpiRNAs in Infected Mosquitoes”).

#### WNV

Midguts of *Cx. pipiens* (*quinquefasciatus*) mosquitoes show a clear RNAi response upon oral infection with WNV (Brackney et al., 2009). The majority of vsiRNAs had a size between 20 and 22 nt with a clear peak of 21 nt. RNAi targeting of the positive and negative sense of the WNV genome was proportional to the abundance of the genome strands. However, clear hot spots for generation of vsiRNAs were observed, most notably a 200 nt region in the 5′-part of the *Capsid* coding sequence, while strongly structured regions in the 3′-UTR were not targeted. Similar profiles of viral small RNAs were also obtained in *Culex* mosquito bodies after oral infection with WNV that, however, also included targeting of the 3′-UTR region (the site of sfRNA production; see also section “SfRNA”; Fros et al., 2015; Göertz et al., 2016). A very recent study reported the detection of 21 nt vsiRNAs in midgut and salivary glands following infection of *Cx. quinquefasciatus* and *Ae. aegypti* mosquitoes (Rückert et al., 2019).

#### ZIKV

*Ae. aegypti* mosquitoes that were orally infected with ZIKV showed a clear exo-RNAi response at 7 and 14 days, i.e., the production of 21 nt vsiRNAs that were distributed across the whole genome but showed a slight bias for the positive strand (analysis in whole mosquitoes; Saldaña et al., 2017). Interestingly, knock-down of *ago-2* in *Ae. aegypti*-derived cells did not result in increased viral replication of ZIKV (Varjak et al., 2017b) while only a minor effect was also demonstrated for DENV replication (Miesen et al., 2016a).

#### Yellow Fever Virus (YFV)

Replication of YFV was demonstrated to be significantly higher in *dcr-2* mutant *Ae. aegypti* (its natural host) compared with wild-type at comparable time points (Samuel et al., 2016). In addition, it was shown that the capsid protein of YFV could act as a VSR in the context of recombinant SINV infections and several biochemical assays (see also section “Evidence for Presence of VSR Genes in Arboviral Genomes” for discussion on VSRs).

### Bunyaviruses

The most studied arboviruses of the Bunyavirales order include Rift Valley Fever virus (RVFV; genus Phlebovirus; *Phenuiviridae* family), La Crosse virus, Schmallenberg virus and the type species Bunyamwera virus (LACV, SBV and BUNV, respectively; Orthobunyavirus genus; *Peribunyavirales* family). Bunyaviruses are characterized by a tripartite linear (-) or ambisense ssRNA genome that is organized in three segments, L (6–7 kb), M (3.0–4.5 kb), and S (1.0–1.7 kb). Several different species of mosquito (e.g., *Ae. aegypti*, *Cx. quinquefaciatus*) are able to act as vectors for transmission of RVFV (Linthicum et al., 2016). LACV is maintained in a cycle between its primary vector, *Ae. triseriatus*, and small mammals, with humans considered as “dead-end” hosts (Beaty et al., 2000). For both RVFV and LACV, vertical transmission to the offspring can occur. The primary vector of BUNV is considered *Ae. aegypti*, although it can also be transmitted by *An. gambiae* but not by *Cx. quinquefasciatus* (Dutuze et al., 2018). Finally, SBV is an arbovirus believed to be transmitted by midge (*Culicoides*) species (Schnettler et al., 2013a).

#### RVFV

Rift Valley Fever virus infection (through infectious blood meal) of three different mosquito species (*Ae. aegypti*, *Ae. vexans*, *Cx. quinquefaciatus*) resulted in the production of vsiRNAs that were derived from all three segments with abundance gradient M > S > L and corresponded to genome and antigenome strands in approximately equal ratios (Dietrich et al., 2017a). A hotspot detected at the intergenic region between the N and Ns genes in the ambisense S segment (observed in mosquito cell lines but to a lesser extent also in *Culex* mosquitoes) could be caused by hybridization of complementary N and NSs mRNAs (Sabin et al., 2013). However, the abundance of viral small RNAs (including vsiRNAs but also vpiRNAs; see section “What is the Role of the piRNA Pathway in Antiviral Defense?”) following infection was observed to be much lower in *Culex* than in *Aedes* mosquitoes (Dietrich et al., 2017a). While this could indicate differences in the RNAi response, other explanations are possible, such as infection status, virus-specific effects or co-infection with mosquito-specific viruses (see also section “RNAi and the Antiviral Defense Against Mosquito-Specific Viruses”).

## Evidence for Presence of VSR Genes in Arboviral Genomes

Initially, the prevailing dogma was that arboviruses do not encode VSRs because of the need to establish long-lasting (7–14 days) persistent infections in mosquito vectors such that transmission to the next vertebrate host can be accomplished. The importance to replicate at low levels for maintenance of the persistent state was dramatically demonstrated in experiments using recombinant SINV that expresses the strong RNAi inhibitor protein B2 of FHV (*Nodaviridae*; Bronkhorst and van Rij, 2014). Injection of SINV-B2 in *Ae. aegypti* mosquitoes resulted in high mortality concomitantly with high levels of viral replication and a greatly reduced RNAi response (production of 21 nt vsiRNAs; Myles et al., 2008). Similar results were obtained in the Aag-2 cell line (derived from *Ae. aegypti*) and during oral infections of mosquitoes with SINV-B2 (Cirimotich et al., 2009). Altogether, these results suggest that at least SINV does not encode a VSR because of the need to maintain the persistent character of the infection. The absence of a VSR gene in alphaviruses was confirmed by the observation that infections of SFV did not affect gene silencing of a reporter construct in the U4.4 mosquito cell line (Attarzadeh-Yazdi et al., 2009). Infection of *An. gambiae* with recombinant ONNV expressing B2 from Nodamura virus (*Nodaviridae*) by both injection and feeding also resulted in increases in mortality and viral titers (Myles et al., 2008).

Recombinant SINV was subsequently used to test candidate VSRs from other viruses, including other arboviruses (Samuel et al., 2016). Using this system, it was established that the capsid protein of YFV (*Flaviviridae*) possesses strong VSR activity. VSR activity could also be demonstrated for capsid proteins of other flaviviruses infecting different hosts (ZIKV and DENV-2 naturally infecting *Ae. aegypti* and humans; WNV infecting *Culex* mosquitoes and birds; Rio Bravo virus (RBV) isolated from bats but with unknown vector) (Samuel et al., 2016). Furthermore, the decreased replication of wild-type SINV compared to recombinant SINV expressing capsid of YFV can be rescued in *Ae. aegypti* mosquitoes that are mutant for *dcr-2*. Biochemical tests demonstrate that the capsid protein of YFV interferes with Dicer processing through binding of long dsRNA (but not siRNA) (Samuel et al., 2016). While no VSR activity could be demonstrated using the recombinant SINV system for other candidates such as subgenomic flaviviral RNA (sfRNA) of WNV, NS4B of DENV or NSs of BUNV, indications of such VSR activity were suggested using other experimental procedures or biochemical assays, as outlined in the following paragraphs.

While alphaviruses (SINV, SFV) are generally not considered to encode VSRs (and actually cause mortality when engineered to express an exogenous RNAi inhibitor; see above), a recent study nevertheless showed that the non-structural proteins nsP2 and nsP3 of CHIKV can inhibit dsRNA- and siRNA-mediated silencing in insect, mammalian and plant cells (Mathur et al., 2016). VSR activity presumably occurs through dsRNA/siRNA binding by RNA-binding motifs present in the helicase domain of nsP2 and the N-terminal macrodomain of nsP3.

### SfRNA

During flaviviral infections, accumulation of sfRNA, an abundant non-coding subgenomic sfRNA representing the last 525 nt of the 3′UTR, is observed as a result of incomplete degradation of the flaviviral genome by the 5′–3′ exoribonuclease XRN1 located in processing bodies (PBs) in mammalian cells, where also the RNAi machinery is located (Pijlman, 2014; Roby et al., 2014). Incomplete degradation is caused by stalling of XRN1 at the 3′-UTR that is characterized by a high degree of secondary structure.

In contrast to the non-structural proteins or the capsid protein of WNV, sfRNA was capable to suppress gene silencing in mammalian cells using reporter assays (Schnettler et al., 2012). Suppression of silencing by sfRNA was also observed in *Ae. albopictus* U4.4 cells and *Drosophila* S2 cells. Engineering of the alphavirus SFV with sfRNA of WNV increased its replication in mosquito cells (in apparent contrast to the absence of effects during recombinant SINV infections of *Ae. aegypti* mosquitoes; see above) while reporter assays established that sfRNA could interfere with both siRNA- and miRNA-mediated silencing in insect cells (Schnettler et al., 2012). In WNV-infected *Cx. pipiens* mosquito strains, sequencing results show that specific hot spots of vsiRNAs derive from structured regions in the 3′-UTR, indicating that sfRNA might be processed by the RNAi machinery *in vivo* (Göertz et al., 2016). However, overall vsiRNA levels and profiles are similar in infections of wild-type WNV and WNV deficient for full-length sfRNA (sfRNA1) production, indicating that sfRNA1 does not interfere with the RNAi response, although unique hot spots of vsiRNAs corresponding to sfRNA1 were identified in infections with wild-type WNV (Göertz et al., 2016).

Interestingly, it was demonstrated that production of sfRNA is a key factor to overcome the midgut barrier in *Culex* mosquitoes during oral infections of WNV (Göertz et al., 2016). On the other hand, transmission and infection rates were not affected for sfRNA-deficient WNV after intrathoracic injection and deficient sfRNA production also did not affect growth rates in mosquito cell lines. It is not clear whether the requirement for sfRNA to overcome the midgut barrier is related to its possible role as a VSR.

Investigations regarding RNAi inhibitory activity of sfRNA were extended to *Cx. quinquefasciatus* mosquitoes infected with either wild-type Kunjin virus (KUNV; *Flaviviridae*) or a mutant form that is defective for production of sfRNA (Moon et al., 2015). While the amount of viral genomic RNA in infected mosquitoes did not differ between wild-type or mutant KUNV (weak) suppression of dsRNA-mediated gene silencing of the endogenous *chymotrypsin* gene was only observed during infection with wild-type KUNV, therefore associating production of sfRNA with inhibition of RNAi. In the same study (Moon et al., 2015), sfRNA was found to be associated with both Dicer and AGO2 in human cells, therefore implicating a role for sfRNA as an RNA decoy for the (human) dsRNA-binding proteins Dicer and AGO2.

Similar experiments demonstrated also suppressor of RNA silencing activity for DENV-1 sfRNA in *Ae. albopictus* U4.4 cells (Schnettler et al., 2012. Furthermore, expression of sfRNAs of tick-borne arboviruses was reported to inhibit RNAi in tick-derived culture cells (Schnettler et al., 2014).

For its proposed VSR activity, it is hypothesized that sfRNA acts as an RNA decoy and inhibits RNAi by oversaturating Dicer enzyme (Pijlman, 2014; Göertz et al., 2016). In mammalian cells, it was indeed demonstrated that siRNAs can bypass the inhibitory effect of sfRNA, indicating that the function of RISC complexes was not affected (Schnettler et al., 2012).

### NS4B

Also in apparent contrast with the results of the recombinant SINV system (Samuel et al., 2016), another study showed that the NS4B protein of DENV could function as a strong RNAi suppressor, in addition to its role in alfa/beta interferon inhibition (Kakumani et al., 2013). While most experiments were carried out using mammalian cells, it is noted that VSR activity of the non-structural protein NS4B was also demonstrated in Sf21 RNAi sensor cells, a transformed insect (lepidopteran) cell line. NS4B is a small transmembrane protein with demonstrated functions involved in RNA replication and interference with the interferon response while the mechanism by which NS4B could inhibit RNAi remains uncharacterized.

### NSs

In mammalian cells, NSs protein from LACV exhibited VSR activity in reporter assays (Soldan et al., 2005). In addition, BUNV that are deficient for NSs exhibited lower replication than wild-type BUNV in a mosquito cell line that is RNAi competent (U4.4; derived from *Ae. albopictus*) while no effect was observed in RNAi-deficient cells (C6/36 and C7/10; also derived from *Ae. albopictus*) (Szemiel et al., 2012) (see also section “Analysis of the Role of the piRNA Pathway in Antiviral Defense in Mosquito Cell Lines” for a discussion of RNAi effects in mosquito cell lines). The exclusive observation of the effect of deletion of NSs on viral replication in the cell line with an intact RNAi mechanism was interpreted as evidence for NSs acting as VSR. However, another study failed to show RNAi suppressor activity for NSs after plasmid-mediated expression in SFV-infected U4.4 cells (Blakqori et al., 2007). In the same study, it was reported that persistent infections of C6/36 cells with LACV resulted in the production of viral small RNAs (the exact size was not determined and these may correspond to piRNAs or other degradation products and not vsiRNAs since C6/36 cells were found later to be Dicer-2 deficient; see section “Dcr-2-Defective *Ae. albopictus* Cell Lines”). Furthermore, persistent infections of wild-type LACV and LACV deficient for NSs showed similar growth properties in C6/36 cells, indicating that NSs did not have a function as a suppressor of innate immunity (while a role as inhibitor of the exo-RNAi pathway could not be addressed in this experiment) (Blakqori et al., 2007). RVFV infection (another bunyavirus that encodes NSs) also did not interfere with RNAi silencing of a reporter gene, indicating the absence of VSR activity (Dietrich et al., 2017a).

### Other Mechanisms of Resistance Against RNAi

Evidence has been presented for selection of genomes with mutations in regions highly targeted by RNAi as a mechanism of evasion (Brackney et al., 2009, 2015; review: Prasad et al., 2013; Blair and Olson, 2014, 2015a,b).

High targeting with vsiRNAs in regions of the genome of WNV could indeed be correlated with increased genetic diversity, indicating a mechanism for evading the RNAi response that restricts WNV replication (Brackney et al., 2009). Analysis of the RNAi response in mosquito bodies after feeding of an infectious blood meal of WNV also indicated differential modulation of viral small RNA profiles in mosquitoes of different *Culex* strains (Göertz et al., 2016), possibly implicating differences in selection pressure for particular virus–host combinations. Interestingly, no differences in the overall pattern of vsiRNAs between wild-type WNV and WNV defective for sfRNA1 (an abundant non-coding viral RNA produced during infection; see section “SfRNA”) were observed, although specific vsiRNAs were identified that are derived from sfRNA1 in wild-type WNV (Göertz et al., 2016).

After passage of WNV in *Drosophila* S2 cells, the number of polymorphic sites was decreased after knock-down of *dcr-2* or *ago-2* while it was increased after targeting the WNV genome with dsRNA (Brackney et al., 2015). RdRp enzymes from RNA viruses have a high error rate which could function as an evolutionary mechanism to escape targeting by vsiRNAs produced during the host antiviral response. Indeed, CHIKV viruses engineered with a high fidelity RdRp show lower infectivity and dissemination rate (Coffey et al., 2011), suggesting that generation of variety in viral genome sequences may be important for efficient infection (see also section “Engineering the RdRP Engines of Arbovirus Replication”).

When the cell lines U4.4 from *Ae. albopictus* and Aag-2 from *Ae. aegypti* were used to analyze viral small RNA production following infection with SFV, accumulation of 21 nt vsiRNAs in hot spots and cold spots along the genome was observed but sequence analysis did not identify any correlation between predicted RNA secondary structure and abundance of vsiRNAs. Interestingly, hot spot-derived vsiRNAs were much less effective in triggering gene silencing as cold spot-derived viRNAs, indicating a possible mechanism of suppression of RNAi through a decoy mechanism (Siu et al., 2011). It has indeed been noticed that the propensity to produce siRNAs because of strong secondary structures can be accompanied by the resistance of those secondary structures to the RISC complex, leading to an evasion of the RNAi response (Fragkoudis et al., 2009).

### Inconclusive Evidence?

While the existence of VSR genes in some arboviruses is suggested, it must be stressed that so far no conclusive proof was obtained that involves mutation of the VSR in the context of the arboviral genome during infections of natural hosts (discussed in O’Neal et al., 2014). Capsid proteins and many other proteins encoded by RNA viruses may have RNA binding activity that is unrelated to VSR activity in the context of viral infection but nevertheless may act as VSRs in other artificial assays, for instance during reporter assays in cell lines and biochemical assays of dicing and slicing.

Also the use of recombinant alphaviruses (SINV or SFV) to test candidate VSRs in infected mosquitoes may result in the identification of false positives. While recombinant SFV expressing capsid protein of ZIKV could replicate to higher levels than control SFV as observed before for recombinant SINV (Samuel et al., 2016), this effect was observed in both wild-type and Dcr-2-knock-out Aag-2 cells (Varjak et al., 2017a), indicating that the effect did not involve the antiviral siRNA (exo-RNAi) pathway. Consistent with this, it was observed that over-expression of capsid protein of ZIKV did not inhibit dsRNA- or siRNA-mediated silencing of a reporter gene in Aag-2 cells (Varjak et al., 2017a). During ZIKV infections of Aag-2 cells, also no inhibition of dsRNA- or siRNA-mediated silencing of a reporter gene was detected (Varjak et al., 2017a).

## What Is the Role of the piRNA Pathway in Antiviral Defense?

In mosquitoes, Argonaute proteins of the PIWI class are expressed in somatic tissues, in contrast to *Drosophila*, where expression is predominantly in the germline (Lewis et al., 2016). Consistent with this observation, piRNAs of viral origin (viral piRNAs or vpiRNAs) were observed in somatic tissues during infections of mosquitoes (Morazzani et al., 2012). In addition, cell lines derived from mosquitoes were used to analyze the contribution of PIWI class proteins and production of vpiRNAs to the antiviral defense (Miesen et al., 2016b). Of significance was also the identification of mosquito cell lines that are defective in the production of vsiRNAs (Scott et al., 2010; Morazzani et al., 2012) which were subsequently employed to demonstrate the involvement of the piRNA pathway in antiviral defense. To directly assess the effect of the Dcr-2 deficiency in Aag-2 cells, a clonal cell line defective in Dcr-2 was engineered using CRISPR-Cas and used to analyze the importance of the piRNA pathway versus the siRNA pathway in antiviral defense (Varjak et al., 2017b).

While piRNAs are defined by their association with Argonaute proteins of the PIWI class (for instance through their identification in specific immunoprecipitates), such information is not available in many studies that describe the generation of “viral small RNAs of piRNA size” or “vpiRNA-like small RNAs.” Using a strict definition, typical hallmarks of piRNAs are: (1) sizes of 25–29 nt; (2) ping-pong amplification signature (U1 antisense, A10 sense); (3) enrichment of the separation of the 5′-ends of complementary viral piRNAs by 10 nt; and (4) resistance to β-elimination indicating 2′-*O-*methylation at the 3′ terminal nucleotide (Vodovar et al., 2012). However, it is also known that piRNAs that are generated in low abundance by the primary pathway (during early infection) will not show a ping-pong signature (Goic et al., 2016; see section “Alphaviruses”). The production of genuine piRNAs may also be obscured by the parallel generation of small RNAs through other degradation pathways. In some studies, “shoulders” of viral small RNAs of piRNA size are present in small RNA profiles that do not have a characteristic sequence logo (e.g., for profiles obtained from flavivirus-infected *Culex* mosquitoes; Fros et al., 2015; Göertz et al., 2016; and alpha virus-infected *An. gambiae*; Carissimo et al., 2015). Whether “viral small RNAs of piRNA size” or “vpiRNA-like small RNAs” in the absence of other characteristic features are produced by a similar piRNA pathway as that documented for the control of transposons (Senti and Brennecke, 2010), still requires further investigation.

### Production of VpiRNAs in Infected Mosquitoes

Below follows a detailed analysis of the occurrence of the piRNA pathway as a potential antiviral defense mechanism in mosquitoes. Production of vpiRNAs is summarized in Table 1. An overview of the possible involvement of factors in the piRNA pathway is presented in Table 2.

#### Alphaviruses

When *Ae. aegypti* and *Ae. albopictus* mosquitoes were injected with CHIKV, not only vsiRNAs (21 nt) were produced but also viral small RNAs of piRNA size (23–30 nt) (Morazzani et al., 2012). VpiRNAs exhibited a strong positive-strand bias and preferentially located on the region of the subgenomic RNA, with clear hotspots. Production of both vsiRNAs and vpiRNAs was also observed during oral infection of *Ae. albopictus* mosquitoes with CHIKV (Goic et al., 2016). During early infection (3 days p.i.), vpiRNA-like molecules (27–29 nt) were not abundant and did not show a sequence bias, in contrast to late infection (9 days p.i.), during which abundant vpiRNAs with ping-pong signature could be detected. The differences may reflect the production of primary vpiRNAs during early infection while abundant secondary vpiRNAs accumulate during late infection by the ping-pong amplification mechanism (Goic et al., 2016).

#### Flaviviruses

During oral infection of *Ae. aegypti* mosquitoes with DENV-2, the presence of piRNA-sized viral small RNAs (24–30 nt) was revealed preferentially during early infection along with the presence of canonical vsiRNAs and viral small RNAs of unusually short length (<20 nt) (Hess et al., 2011). After a decrease in viral small RNA levels at 4 days post infection, much higher levels of viral small RNAs were observed during late infection which corresponded mostly to vsiRNAs (20–23 nt). Viral small RNAs of piRNA size were preferentially of sense orientation and showed a weak signature of enrichment of adenine at the 10th base (A10) while no bias for the presence of uridine at position 1 (1U) was observed (Hess et al., 2011). It is noted that the preferential early accumulation of vpiRNAs during DENV-2 infection contrasts with their higher presence during later periods of infection by CHIKV (see above; Goic et al., 2016).

Similarly, after oral infection of *Ae. albopictus* mosquitoes with DENV-2, three classes of viral small RNAs could be identified: unusually small (ultrashort) RNAs (usRNAs; 13–19 nt), vsiRNAs (20–23 nt) and candidate vpiRNAs (24–30 nt) (Wang et al., 2018). Both vsiRNAs and piRNA-like small viral RNAs showed a strong positive-strand bias. A clear ping-pong signature was not detected, since the 1U bias for the antisense reads was absent while only a weak preference for A10 in the sense reads was observed; furthermore, no 10 nt overlap between sense and antisense reads was detected. Interestingly, differences in viral small RNA accumulation occurred in whole bodies and midguts of female adult mosquitoes: vsiRNAs were predominant in whole body samples while both candidate vpiRNAs and usRNAs were much more prevalent in midgut. In libraries from whole bodies of adult females, piRNA-like small viral RNAs derived mainly from a few hotspots in the DENV2 genome, located at the non-structural protein 5 (NS5) region, while a more broad distribution was observed for piRNA-like viral small RNAs across the DENV-2 genome in libraries of adult female midguts (Wang et al., 2018).

In *Ae. albopictus*, expression of *piwi-1-4* is increased in adult females after blood feeding. However, no increase in expression of *ago-3* and Piwi-related genes is observed following DENV-2 infection of adult females (Wang et al., 2018). During infection of *Culex*-derived cell lines with WNV, only production of vsiRNAs was observed and no induction in the expression of Piwi-related and *ago-3* genes was detected (Rückert et al., 2019).

Besides the production of 21 nt vsiRNAs, oral infection of *Ae. aegypti* mosquitoes with ZIKV also resulted in the production of 25–30 nt viral small RNAs (Saldaña et al., 2017). However, because no ping-pong signature (U1, A10) was observed, the viral small RNAs, which were almost exclusively derived from the positive strand, may have been produced by other RNA degradation pathways and therefore could not be reliably identified as vpiRNAs.

#### Bunyaviruses

Rift Valley Fever virus infection (through infectious blood meal) of three different mosquito species (*Ae. aegypti*, *Ae. vexans*, *Culex quinquefaciatus*) resulted in the production of both vsiRNAs and vpiRNAs (Dietrich et al., 2017a). However, much lower levels of vsiRNAs and vpiRNAs were detected during infection of *Culex* mosquitoes. VpiRNAs of 26–30 nt that were detected displayed the characteristic ping-pong signature (sequence bias of 1U for genome strands and A10 for antigenome strands; 10 nt overlap between 5′-ends of complementary strands of small RNAs). Clear strand enrichment was observed for RVFV-derived vpiRNAs but differed according to the different segments (M and S: antigenome bias; L: genome bias). For the S segment, the majority of (sense, antigenome) vpiRNAs were derived from the region of the mRNA encoding the nucleocapsid (N) protein (the other gene of the ambisense S segment (NSs) being transcribed from the antigenome strand; Dietrich et al., 2017a).

#### vpiRNAs in *Culex* Mosquitoes

The abundance of viral small RNAs following infection was observed to be much lower in *Culex* than in *Aedes* mosquitoes although this could be caused by infection status or virus-specific effects (Dietrich et al., 2017a). For *Culex* mosquitoes, low amounts of vpiRNAs were produced during infection with RVFV (Bunyavirales) (Dietrich et al., 2017a) while they were not reported during WNV or Usutu virus (USUV) (*Flaviviridae*) infection (Brackney et al., 2009; Fros et al., 2015; Göertz et al., 2016) or SINV (Alphavirus) infection (Miesen et al., 2016b). Although the relative importance of the contribution still needs to be investigated in detail, the detection of RVFV-specific vpiRNAs suggests that the possibility of contribution of the piRNA pathway to the antiviral defense can be extended to *Culex* mosquitoes.

### Analysis of the Role of the piRNA Pathway in Antiviral Defense in Mosquito Cell Lines

Research on the significance of the piRNA pathway in antiviral defense has benefited from the availability of *Ae. albopictus* cell lines (C6/36 and C7-10) that are deficient in the production of vsiRNAs and that show higher production of viral titers in comparison with mosquito cell lines that are competent for vsiRNA production [Aag-2 (*Ae. aegypti*); U4.4 (*Ae. albopictus*)] (Scott et al., 2010; Morazzani et al., 2012). Another important line of research employed the Aag-2 cell line from *Ae. aegypti* in which relatively efficient knock-down of components of the piRNA- and siRNA-pathway could be achieved in order to evaluate their contribution to antiviral defense (Miesen et al., 2015). In these studies, production of vsiRNAs and vpiRNAs as well as the involvement of components of the siRNA and piRNA pathways are often directly compared.

Comparable results with respect to vsiRNA and vpiRNA production as in U4.4 and Aag-2 cell lines were also obtained with the TRA-171 cell line from the predatory mosquito *Toxorhynchites amboinensis* (Culicidae) infected with SFV (*Togaviridae*) (Donald et al., 2018). By contrast, no canonical vpiRNAs were observed after infection of the KC cell line of the midge *Cu. sonorensis* with Bluetongue virus (*Reoviridae*), SBV or BUNV (both Bunyavirales) (Schnettler et al., 2013b; Dietrich et al., 2017b).

Below follows a detailed analysis of the occurrence of the piRNA pathway as a potential antiviral defense mechanism in mosquito cell lines. Comparison of the significance of the piRNA pathway with the exo-RNAi pathway regarding antiviral defense is provided. Production of vpiRNAs and vsiRNAs in cell lines is summarized in Table 3. An overview of the possible involvement of factors in the piRNA pathway (together with the exo-RNAi and miRNA pathways) in antiviral defense in the Aag-2 cell line is presented in Table 4.

**Table 3:** Viral small RNAs produced during arbovirus infections of mosquito cell lines.

Virus	Cell line	Viral small RNAs	References
***Alphavirus***			
CHIKV	C6/36 (*Ae. albopictus*) C7/10 (*Ae. albopictus*) (both Dcr-2-defective)	23–30 nt vpiRNA	Morazzani et al., 2012
CHIKV	C6/36 (*Ae. albopictus*) (Dcr-2-defective)	25–30 nt vpiRNA	Goic et al., 2016
CHIKV	U4.4 (*Ae. albopictus*)	21 nt vsiRNA 25–29 nt vpiRNA	Morazzani et al., 2012
SFV	Aag-2 (*Ae. aegypti*)	21 nt vsiRNA 25–29 nt vpiRNA	Schnettler et al., 2013a
SFV	AF5 clone (Aag-2) (*Ae. aegypti*)	21 nt vsiRA 26–29 nt vpiRNA	Varjak et al., 2017b
SFV	AF319 Dcr-2 KO (Aag-2) (*Ae. aegypti*)	21 nt vsiRA 25–32 nt vpiRNA	Varjak et al., 2017b
SFV	U4.4 (*Ae. albopictus*)	21 nt vsiRNA 25–29 nt vpiRNA	Schnettler et al., 2013a
SFV	TRA-171 (*Toxorhynchites amboinensis*)	21 nt vsiRNA 26–29 nt vpiRNA	Donald et al., 2018
SINV	C6/36 (*Ae. albopictus*) (Dcr-2-defective)	23–28 nt vpiRNA	Brackney et al., 2010
SINV	Aag-2 (*Ae. aegypti*)	21 nt vsiRNA 25–29 nt vpiRNA	Vodovar et al., 2012 Miesen et al., 2015
SINV	U4.4 (*Ae. albopictus*)	21 nt vsiRNA 25–29 nt vpiRNA	Vodovar et al., 2012
***Flavivirus***			
DENV	C6/36 (*Ae. albopictus*) (Dcr-2-defective)	27 nt vpiRNA	Scott et al., 2010
DENV	Aag-2	21 nt vsiRNA 25–30 nt vpiRNA	Miesen et al., 2016a
WNV	C6/36 (*Ae. albopictus*) (Dcr-2-defective)	19–20 nt usRNA	Brackney et al., 2010
WNV	Hsu (*Cx. quinquefasciatus*)	21 nt vsiRNA	Rückert et al., 2019
WNV	CT (*Cx. tarsalis*)	21 nt vsiRNA	Rückert et al., 2019
ZIKV	Aag-2	20–21 nt vsiRNA 25–28 nt vpiRNA	Varjak et al., 2017a
**Bunyaviridae**			
BUNV	Aag-2 (*Ae. aegypti*)	14–19 nt usRNA 21 nt vsiRNA 25–28 nt vpiRNA	Dietrich et al., 2017b
BUNV	U4.4 (*Ae. albopictus*)	15–17 nt usRNA 21 nt vsiRNA 26–30 nt vpiRNA	Dietrich et al., 2017b
BUNV	KC (*Culicoides sonorensis*)	15–17 nt usRNA 21 nt vsiRNA 26–29 nt vpiRNA	Dietrich et al., 2017b
LACV	C6/36 (*Ae. albopictus*) (Dcr-2-defective)	24–28 nt vpiRNA	Brackney et al., 2010
LACV	C6/36 (*Ae. albopictus*) (Dcr-2-defective)	25–29 nt vpiRNA	Vodovar et al., 2012
RVFV	C6/36 (*Ae. albopictus*) (Dcr-2-defective)	14–19 nt usRNA 24–29 nt vpiRNA	Léger et al., 2013
RVFV	Aag-2 (*Ae. aegypti*)	14–19 nt usRNA 21 nt vsiRNA 25–28 nt vpiRNA	Léger et al., 2013
RVFV	Aag-2 (*Ae. aegypti*)	21 nt vsiRNA 24–32 nt vpiRNA	Dietrich et al., 2017a
RVFV	U4.4 (*Ae. albopictus*)	14–19 nt usRNA 21 nt vsiRNA 25–28 nt vpiRNA	Léger et al., 2013
SBV	Aag-2 (*Ae. aegypti*)	15–17 nt usRNA 21 nt vsiRNA 25–30 nt vpiRNA	Schnettler et al., 2013b Dietrich et al., 2017b
**Reoviridae**			
BTV	Aag-2 (*Ae. aegypti*)	21 nt vsiRNA 25–33 nt vpiRNA	Schnettler et al., 2013b


**Table 4:** RNAi factors that affect arbovirus replication in Aag-2 (*Ae. aegypti*) cell line.

Virus	RNAi factor	Phenotype after knock-down	References
***Alphavirus***			
CHIKV	Ago-2	Increased replication (replicon) Increased viral replication	McFarlane et al., 2014 Varjak et al., 2018a
CHIKV	Ago-3	Increased viral replication (minor effect) Decreased production of vpiRNAs	Varjak et al., 2018a
CHIKV	Piwi-4	Increased viral replication	Varjak et al., 2018a
CHIKV	Piwi-5	Decreased production of vpiRNAs	Varjak et al., 2018a
CHIKV	SpnE	Increased viral replication Decreased production of vpiRNAs (minor effect)	Varjak et al., 2018a
SFV	Ago-2	Increased viral replication	Schnettler et al., 2013a; Varjak et al., 2017a
SFV	Ago-2	Increased viral replication in AF5 (Dcr-2 competent) More pronounced than in AF319 (Dcr-2 defective)	Varjak et al., 2017b
SFV	Ago-3	Increased viral replication (minor effect)	Varjak et al., 2017a
SFV	Ago-3	Decreased production of vpiRNAs	Varjak et al., 2018a
SFV	all Piwi/Ago-3	No production of vpiRNAs	Schnettler et al., 2013a
SFV	Piwi-4	Increased viral replication	Schnettler et al., 2013a; Varjak et al., 2017a, 2018a
SFV	Piwi-4	Increased viral replication with similar efficiency in both AF5(Dcr-2 competent) and AF319 (Dcr-2 defective)	Varjak et al., 2017b
SFV	Piwi-5	Decreased production of vpiRNAs	Varjak et al., 2018a
SFV	Piwi-6	Decreased production of vpiRNAs (minor effect)	Varjak et al., 2018a
SFV	SpnE	Increased viral replication Decreased production of vpiRNAs (minor effect)	Varjak et al., 2018a
SINV	Piwi-4	Decreased production of vpiRNAs (minor effect) Increased production of vsiRNAs	Miesen et al., 2015
SINV	Piwi-5	Decreased production of vpiRNAs Increased production of vsiRNAs	Miesen et al., 2015
SINV	Ago-3	Decreased production of vpiRNAs	Miesen et al., 2015
***Flavivirus***			
DENV	Ago-3	Decreased production of vpiRNAs	Miesen et al., 2016a
DENV	Piwi-5	Decreased production of vpiRNAs	Miesen et al., 2016a
DENV	Piwi-6	Decreased production of vpiRNAs (minor effect)	Miesen et al., 2016a
ZIKV	Dcr-2	Increased viral replication	Varjak et al., 2017a
ZIKV	Ago-2	Decreased viral replication (proviral)	Varjak et al., 2017a
ZIKV	Ago-3	Decreased viral replication (proviral)	Varjak et al., 2017a
ZIKV	Piwi-4	Increased viral replication	Varjak et al., 2017a
**Bunyaviridae**			
BUNV	Ago-1	Decreased viral replication (proviral)	Dietrich et al., 2017b
BUNV	Ago-2	Increased viral replication	Dietrich et al., 2017b
BUNV	Ago-3	Decreased viral replication (proviral)	Dietrich et al., 2017b
BUNV	Piwi-4	Increased viral replication	Dietrich et al., 2017b
BUNV	Piwi-6	Decreased viral replication (proviral)	Dietrich et al., 2017b
CVV	Ago-1	Decreased viral replication (proviral)	Dietrich et al., 2017b
CVV	Ago-2	Increased viral replication	Dietrich et al., 2017b
CVV	Piwi-4	Increased viral replication	Dietrich et al., 2017b
SATV	Ago-1	Increased viral replication	Dietrich et al., 2017b
SATV	Ago-2	Increased viral replication	Dietrich et al., 2017b
SBV	Ago-1	Increased viral replication	Dietrich et al., 2017b
SBV	Ago-2	Increased viral replication	Dietrich et al., 2017b
SBV	Piwi-5	Decreased viral replication (proviral)	Dietrich et al., 2017b
RVFV	Ago-2	Increased viral replication	Dietrich et al., 2017a
RVFV	Ago-3	Increased viral replication	Dietrich et al., 2017a
RVFV	Piwi-4	Increased viral replication	Dietrich et al., 2017a


#### Dcr-2-Defective *Ae. albopictus* Cell Lines

During RNA virus infection of the C6/36 cell line from *Ae. albopictus*, non-canonical patterns of viral small RNAs are observed that are likely to be caused by its inability of dicing dsRNA substrates and a non-functional Dcr-2 enzyme (Scott et al., 2010). Genotyping revealed a homozygous frameshift mutation in the ORF of *dcr-2* in C6/36 cells resulting in the formation of a premature stop codon (Morazzani et al., 2012). Another cell line of *Ae. albopictus*, C7-10, was also reported to be detective in the siRNA pathway, caused by a deletion of 33 AA between the DUF and PAZ domains of Dcr-2 (Morazzani et al., 2012). In several studies, the antiviral RNAi response in Dcr-2-deficient C6/36 and C7-10 cell lines was compared with the response in Dcr-2-competent cell lines such as Aag-2 (*Ae. aegypti*) and U4.4 (*Ae. albopictus*).

##### Alphavirus

During SINV infections of C6/36 cells mainly viral small RNAs of piRNA size (23–28 nt) were produced that were distributed unevenly between genomic (70%) and antigenomic (30%) strands (Brackney et al., 2010). Hot spots of piRNA-sized small RNAs were observed in the subgenomic region that encodes the structural genes. Similarly, during infection of C6/36 and C7-10 cells with CHIKV (Alphavirus), only vpiRNAs of 23–30 nt size were detected that showed a clear ping-pong amplification (1U antisense, A10 sense) signature (Morazzani et al., 2012; Goic et al., 2016). By contrast, both vsiRNAs and vpiRNAs were produced during CHIKV infection in Dcr-2-competent U4.4 cells (Morazzani et al., 2012; Goic et al., 2016).

##### Flavivirus

During DENV-2 infections of C6/36 cells, viral small RNAs were generated that corresponded almost exclusively from the sense strand and were derived from a few specific regions of the genome (Scott et al., 2010). Moreover, the viral small RNAs had a size of 27 nt and an enrichment of adenine at position 10 (A10) which indicates that they were generated by the piRNA pathway (Scott et al., 2010). On the other hand, infections with another flavivirus, WNV, resulted in the production of a high proportion of viral small RNAs of 17–18 nt that may correspond to degradation products by degradation pathways that are not related to RNAi. The vast majority of viral small RNAs during WNV infection of C6/36 cells were of sense polarity while no viral small RNAs of piRNA size were detected (Brackney et al., 2010).

##### Bunyavirales

Analysis of viral small RNAs produced during infections of C6/36 cells with LACV also revealed the predominant presence of viral small RNAs of piRNA-like size (24–28 nt) (Brackney et al., 2010; Vodovar et al., 2012). Despite LACV being a virus with a segmented negative-sense RNA genome, viral small RNAs from the positive-sense strand (also corresponding to the mRNA) were predominant (>70%) although differences among the three segments were observed (Vodovar et al., 2012). The S segment was much more targeted (by 10-fold) than the M and L segments which can be correlated with the much higher abundance of the S segment mRNA compared to the mRNAs of the other segments (Brackney et al., 2010). LACV-derived small RNAs in C6/36 cells are clearly produced by the piRNA pathway since they show a ping-pong amplification signature (U1 antisense, A10 sense) and the 5′-ends of complementary small RNAs are preferentially separated by 10 nt (Vodovar et al., 2012). Regarding the biogenesis of LACV-derived piRNAs, it can be hypothesized that abundant mRNAs are initially targeted to produce primary (sense) piRNAs, which will subsequently target antisense genomic strands. Viral piRNA production from the genomic strands, however, will be limited because of their low abundance. On the other hand, the few vpiRNAs of antisense orientation from the second step presumably can generate abundant vpiRNAs from the abundant positive strand RNAs (i.e., mRNAs) that are present in the infected cells (Vodovar et al., 2012).

A comparative study was carried out among Dicer-2-competent U4.4 and Aag-2 cell lines and the Dcr-2-defective C6/36 cell line with respect to RVFV infection (ZH548 strain; Léger et al., 2013). Interestingly, persistent infections were only obtained with U4.4 and Aag-2 cells which could be correlated with the clearance of viral NSs filaments from the nuclei of the infected cells. In infections of mammalian cells, the non-structural NSs protein also forms nuclear filaments and plays a fundamental role in the mechanism of pathogenicity, i.e., by interference with cellular transcription. VpiRNAs (27–28 nt) with ping-pong signature were observed after RVFV infection of C6/36 cells, besides the presence of 24–25 nt and 21 nt viral small RNAs that did not show a ping-pong signature. Characteristic of RVFV infections of C6/36 cells (but also observed in Aag-2 and U4.4 cells) was also the production of highly abundant very small viral RNAs (14–19 nt) that are probably generated by another degradation pathway, a phenomenon also observed during WNV infection of C6/36 cells (Brackney et al., 2010; see section “Flavivirus”). While persistent infections of C6/36 cells with RVFV were not obtained (infected C6/36 cells could not be passaged), it was nevertheless observed that infection with a particular RVFV strain could protect against secondary infections (“super-infections”) of a different strain of RVFV in this cell line. These data were interpreted to suggest that functional Dcr-2 is not required for pathogen-derived resistance and that the piRNA pathway alone can support some antiviral response.

#### Knock-Down of Components of piRNA Pathway in Aag-2 Cells

Aag-2, an *Ae. aegypti* cell line of embryonic origin, has been used recently more frequently as a model for immunity studies representative of mosquitoes (Barletta et al., 2012). Besides the genes of the miRNA and siRNA pathways, Aag-2 cells also express the PIWI class Argonaute proteins that are present in somatic tissues of adult mosquitoes (*piwi-4*, *piwi-5*, *piwi-6* and *ago-3*; but not *piwi-1-3* or *piwi-7*; Miesen et al., 2015). However, Aag-2 cells are also persistently infected with the mosquito-specific viruses Phasi-Charoen Like virus (PCLV; Bunyavirales) and cell-fusing agent virus (CFAV; *Flaviviridae*) (Weger-Lucarelli et al., 2018) (see also section “RNAi and the Antiviral Defense Against Mosquito-Specific Viruses”), that may affect arbovirus infections in this cell line (Zhang et al., 2017; Schultz et al., 2018). The impact of mosquito-specific virus infections to modulate concomitant arbovirus infections in mosquitoes is an active area of research (Fredericks et al., 2019).

##### Alphavirus

Infections of Aag-2 cells with alphavirus (SINV, SFV, CHIKV) did not only result in production of vsiRNAs but also of viral small RNAs that have the characteristics of piRNAs (Morazzani et al., 2012; Vodovar et al., 2012; Schnettler et al., 2013a; Miesen et al., 2015). VpiRNAs were mainly of sense orientation, were mostly derived from regions at the subgenomic RNA and showed the distinctive signs of ping-pong amplification (U1 antisense and A10 sense; 10 nt overlap between 5′-termini of complementary reads).

Knock-down of individual Piwi genes established the involvement of cytoplasmic Piwi-5 and Ago-3 in the biogenesis of SINV-derived vpiRNAs in Aag-2 cells. Sequencing of vpiRNAs in immunoprecipitates reveals association of sense vpiRNAs of A10 bias with Ago-3, while antisense vpiRNAs of U1 logo are preferentially bound to Piwi-5 and Piwi-6 (Miesen et al., 2015). On the other hand, Piwi-4 was not found to be associated with any vpiRNAs. Although knock-down of *piwi-4* affected accumulation of piRNAs derived from transposable elements, no piRNAs corresponding to transposon sequences were detected in Piwi-4 immunoprecipitates, leading to the proposal that Piwi-4 indirectly regulates the function of the piRNA pathway (i.e., in the absence of direct binding of piRNAs), possibly by modulating the activity of the other piRNA factors or regulating the amount of substrate that enters the piRNA pathway. The diversification of Piwi-related factors in *Ae. aegypti* mosquitoes likely reflects their specialization in the control of different classes of parasitic nucleic acids (mainly transposons), with Piwi-5 and Ago-3 involved more specifically in production of piRNAs during viral infections (Miesen et al., 2015).

Knock-down experiments of Piwi-related genes in Aag-2 cells also revealed increased SFV replication following silencing of *piwi-4* (as well as *ago-2*). Knock-down of all expressed Piwi-related proteins (*piwi-2*, *piwi-3*, *piwi-4*, *piwi-5*, *piwi-6*, *piwi-7* and *ago-3*) resulted in a strong decrease in production of vpiRNAs during SFV infection. However, knock-down of *piwi-4* had no effect on vpiRNA accumulation, indicating an effector function of Piwi-4 to target and silence the virus independent of vpiRNAs (Schnettler et al., 2013a).

A clonal cell line derived from Aag-2 cells (AF5) was used to engineer a Dcr-2-defective cell line (AF319) representative of *Ae. aegypti* by CRISPR-Cas9 mediated engineering (Varjak et al., 2017b). When infected with SFV, production of vsiRNAs was abolished in AF319 cells while the levels of vpiRNAs were greatly enhanced which correlated with increased viral replication compared to AF5 cells (as also observed for Dicer-defective C6/36 and C7/10 cells derived from *Ae. albopictus*). Despite differences in vpiRNA levels, the properties of SFV-derived vpiRNAs were very similar between Dcr-2-deficient AF319 and Dcr-2-competent AF5 cells such as distribution along the viral genome and the occurrence of the ping-pong signature. Re-introduction of Dcr-2 by expression plasmids also did not alter the properties of vpiRNAs in AF319 cells (Varjak et al., 2017b). Knock-down of *ago-2* had a much reduced effect on SFV replication in AF319 cells, in comparison with AF5 cells, confirming the dependence of the antiviral effects of Ago-2 on production of vsiRNAs produced by Dcr-2. On the other hand, silencing of *piwi-4* increased viral replication in both AF5 and AF319 cells, indicating that the antiviral effect of Piwi-4 does not require Dcr-2. In pull-down experiments using extracts from Dcr-2-deficient AF319 cells (infected with SFV), vpiRNAs with ping-pong signature (but without positive strand bias as is commonly observed) were detected in association with both Ago-2 and Piwi-4. However, binding may be indirect since Piwi-4 is found in complexes with proteins of both siRNA (Ago-2, Dcr-2) and piRNA (Piwi-5, Piwi-6, Ago-3) pathways (Varjak et al., 2017b; see also section “Possible Cross-Talk Between siRNA and piRNA Pathway?”). Furthermore, silencing of *piwi-5* or *ago-3* (both previously shown to be involved in vpiRNA production in Aag-2 cells; Miesen et al., 2015, 2016a) in AF319 cells also did not result in increased SFV replication. The experiments indicate strongly that Piwi-4 is an antiviral effector that is independent of vsiRNA and vpiRNA production. On the other hand, vpiRNAs do not seem to play an antiviral role since they seem to be incapable to replace the role of vsiRNAs in Dcr-2-deficient cells.

Following the demonstration that knock-down of *ago-2* resulted in increased expression of a CHIKV reporter replicon (McFarlane et al., 2014), similar knock-down studies were carried out for *ago-3* and Piwi-related genes which showed a clear antiviral effect for *piwi-4* while the effect for *ago-3* was minor (Varjak et al., 2018a). No antiviral role was revealed for *piwi-5* or *piwi-6*. As for SINV and SFV, knock-down of *ago-3* and *piwi-5* and, to a lesser extent, *piwi-6*, resulted in decreased vpiRNA levels during CHIKV infection.

Knock-down of other factors of the piRNA pathway revealed the antiviral activity of the helicase Spindle-E (SpnE), that was identified as a cofactor in the ping-pong amplification loop in *Drosophila* (Varjak et al., 2018a). No antiviral role was uncovered during silencing of other potential cofactors in the piRNA pathway of mosquitoes (Qin, Vasa, Zucchini, Armitage, GasZ, Hen1 and factors involved in heterochromatin silencing). Knock-down of *SpnE* also resulted in partial suppression of vpiRNAs, similar to knock-down of *piwi-6*. However, the antiviral effect of *SpnE* could not be correlated with the production of both vsiRNAs and vpiRNAs and therefore may be related to its more general role in the regulation of RNA metabolism. Interestingly, the antiviral role of *SpnE* seems to be limited to alphaviruses (SFV, CHIKV) since its knock-down did not affect BUNV (Bunyavirales) or ZIKV (*Flaviviridae*) replication (Varjak et al., 2018a).

##### Flavivirus

During DENV-2 infection of Aag-2 cells, abundant vpiRNAs were also detected in addition to the production of vsiRNAs (Miesen et al., 2016a). DENV-derived vpiRNAs in Aag-2 cells have a characteristic size of 25–30 nt, are resistant to β-elimination (and therefore likely 2′-*O*-methylated at their 3′ termini), show an almost exclusively sense orientation and derive from a very limited number of hotspots (85% of reads correspond to just four vpiRNA sequences; Miesen et al., 2016a). Gene silencing studies in Aag-2 cells reveals the dependence of DENV-derived piRNAs on *ago-3*, *piwi-5* and (partially) *piwi-6*. However, none of the knock-downs of *ago-3* and Piwi-related genes (as well as *ago-2*) resulted in an increase in DENV replication levels in Aag-2 cells (Miesen et al., 2016a).

Infection of Aag-2 cells with ZIKV resulted in production of both vsiRNAs and vpiRNAs (Varjak et al., 2017a). VpiRNA-like molecules (25–29 nt) were derived from a hotspot in the NS5 region at a similar position of the subgenomic RNA as observed during DENV infections. However, no ping-pong signature was observed in the piRNA-like viral small RNAs. Immunoprecipitation experiments showed association of vsiRNAs with Ago-2 and the association of vpiRNA-like molecules with Ago-3 but not Piwi-5 or Piwi-6 during ZIKV infections (Varjak et al., 2017a). In immunoprecipitates of Piwi-4, relatively small amounts of both vsiRNAs and vpiRNA-like small RNAs were detected (but see also section “Possible Cross-Talk Between siRNA and piRNA Pathway?”). Silencing of *piwi-4* resulted in an increase of replication of ZIKV reporter virus in Aag-2 cells, in contrast to silencing of *piwi-5* and *piwi-6*. Possible proviral functions of *ago-2* and *ago-3* were also revealed during infections with ZIKV in Aag-2 cells (Varjak et al., 2017a). Interestingly, an antiviral function of *dcr-2* could be demonstrated during ZIKV infection, in contrast to *ago-2*.

##### Bunyavirales

With respect to the accumulation of viral small RNAs during RVFV infection, it was observed that during the early phases of acute infection, vsiRNAs predominate in Aag-2 and U4.4 cells while during later phases of acute infection and during persistent infection, vpiRNAs became more important (Léger et al., 2013; also observed during alphavirus infection; Goic et al., 2016). While vsiRNAs mapped with roughly equal proportion to genomic and antigenomic strands for the L and M segments, a strong bias for the antigenomic (coding) strand was observed for the vpiRNAs, especially for the S segment, which is known to produce abundant mRNAs. VpiRNAs showed a clear ping-pong signature, with enrichment for U1 and A10 on the genomic (antisense) and antigenomic (sense) strands, respectively. Ping-pong signature was more evident for 27–28 nt than for 24–25 nt viral small RNAs but could also be detected in 21 nt reads during persistent infections of Aag-2 cells.

Viral small RNAs produced during RVFV infection of Aag-2 cells were functional since silencing of reporter constructs with RVFV target sequences was observed during RVFV infection of Aag-2 cells. RVFV could also be targeted by the RNAi machinery after transfection of dsRNA targeting the S and L segments. Furthermore, enhanced RVFV replication was observed after silencing of *ago-2*, *piwi-4* and *ago-3* (but not *piwi-5* or *piwi-6*) in Aag-2 cells, implicating the involvement of both the siRNA and the piRNA pathway in antiviral defense (Dietrich et al., 2017a).

Also infections of SBV (with *Culicoides* midges as natural vectors/hosts) caused production of both vsiRNAs and vpiRNAs in non-host Aag-2 cells (Schnettler et al., 2013b). As for LACV and RVFV, vsiRNAs were equally presented between genomic and antigenomic segments while vpiRNAs were mainly derived from the antigenomic (sense) strand of the S segment. In addition, viral small RNAs of very small size (15–17 nt) were produced, mainly from segments M and L. In infected Aag-2 cells, SBV-derived small RNAs of sizes 24–30 nt displayed characteristics of piRNAs such as a ping-pong signature (U1 antisense, A10 sense) and the preferential separation of 5′-termini of complementary reads by 10 nt (Schnettler et al., 2013b).

Infections of Aag-2 cells (and U4.4 cells) with BUNV resulted in production of both vsiRNAs and vpiRNAs (Dietrich et al., 2017b). VsiRNAs of 21 nt length were predominant for the L segment and mapped to both genome and antigenome in a hot spot and cold spot pattern. VpiRNAs of 24–30 nt length were the major species for the M and S segments with a bias for the genome.

Silencing of different Argonaute and Piwi-related genes in Aag-2 cells revealed different genetic requirements for antiviral defense against mosquito-borne [BUNV and Cache Valley virus (CVV)] and midge-borne bunyaviruses [SBV and Sathuperi virus (SATV)]. While *ago-2* provided antiviral activity to all infections, *piwi-4* only protected against mosquito-borne bunyaviruses (Dietrich et al., 2017b). Interestingly, proviral functions of *ago-3* and *piwi-6* were revealed for infections with mosquito-specific BUNV and of *piwi-5* for (midge-specific) SBV infections. The analysis therefore supports the adaptation of the piRNA pathway in Aag-2 cells to specific vector-virus combinations. The miRNA pathway also has a proviral function during infections with mosquito-specific bunyaviruses, while silencing of *ago-1* increases replication of midge-specific viruses (antiviral function; Dietrich et al., 2017b).

### Possible Cross-Talk Between siRNA and piRNA Pathway?

There are several indications of interactions between siRNA and piRNA pathways during antiviral defense in mosquitoes which were revealed in experiments with recombinant viruses expressing the B2 VSR that sequesters dsRNA and also by the detection of protein complexes that contain factors of both RNAi pathways and their respective small RNAs. During viral infection temporal patterns of vpiRNA and vsiRNA accumulation were also observed suggesting the predominance of a particular pathway at different stages of infection.

Infection of *Ae. albopictus* mosquitoes with recombinant CHIKV that expresses the B2 VSR protein (CHIKV-B2) resulted in higher virulence/mortality and decreased production of vsiRNAs while levels of vpiRNAs were increased (Morazzani et al., 2012; see also section “Evidence for Presence of VSR Genes in Arboviral Genomes” for discussion of VSRs). The capacity of the dsRNA-binding protein B2 to influence both vsiRNA and vpiRNA pathways may indicate that both pathways are initially activated by viral dsRNAs acting as pathogen-associated molecular patterns (PAMPs). In Dcr-2-defective C6/36 cells, infection by CHIKV-B2 caused higher virulence, decreased production of vpiRNAs and a relative increase in the number of viral genomes compared to wild-type CHIKV or CHIKV expressing mutant B2 (Morazzani et al., 2012). The involvement of the dsRNA-binding protein B2 again suggested the possibility that dsRNA plays a role as a PAMP to activate the antiviral piRNA pathway in Dcr2-defective *Ae. albopictus* cell lines (Morazzani et al., 2012). However, low levels of vsiRNAs are also produced during CHIKV infection of C6/36 cells (Goic et al., 2016) by an unknown mechanism (e.g., persistent low activity of Dcr-2 or ectopic activity of Dcr-1) and B2 could therefore act through further inhibition of vsiRNA production. Furthermore, transfection of *luciferase* dsRNA can inhibit replication of the corresponding reporter replicon of WNV (Pitaluga et al., 2008), indicating the presence of a partially active exo-RNAi pathway in C6/36 cells. As previously mentioned, other data, using Dcr-2-deficient Aag-2 cells, indicate the independence of the piRNA pathway from the antiviral siRNA pathway and question the role of vpiRNAs during antiviral defense (Varjak et al., 2017b). When dsRNA is transfected into Aag-2 and U4.4 cells, 21 nt siRNAs but no vpiRNAs are produced, providing further evidence that dsRNA preferentially/exclusively activates the dsRNA pathway (Schnettler et al., 2013a).

During infection of Aag-2 cells by alphavirus, the presence of both vsiRNAs and vpiRNAs was revealed in Piwi-4 immunoprecipitates although the levels were rather low (Varjak et al., 2017b). For both SINV and SFV infections, vpiRNAs with ping-pong characteristics were also found in immunoprecipitates of Ago-2. In addition, it was demonstrated that Piwi-4 can exist in complexes with other proteins of both siRNA (Ago-2, Dcr-2) and piRNA (Piwi-5, Piwi-6, Ago-3) pathways. It is therefore unknown whether viral small RNAs detected in Piwi-4-immunoprecipitates correspond to direct binding to Piwi-4 or to binding to other interacting proteins (Varjak et al., 2017b). While these data point to physical interactions between piRNA- and siRNA-associated Argonaute proteins during antiviral defense, its functional significance remains to be elucidated.

In several cases, differential temporal accumulation of vsiRNAs and vpiRNAs during arboviral infection was reported. Moreover, the preferential generation of vsiRNAs versus vpiRNAs and vice versa over time may depend on the virus and the infection system used (systemic infection of cell lines versus oral infection of mosquitoes). During oral infection of *Ae. aegypti* with DENV, vpiRNAs predominate during early infection while vsiRNAs become much more abundant during late infection (Hess et al., 2011). In Aag-2 and U4.4 cells that were infected by RVFV, by contrast, early stages of acute infection are mainly associated with vsiRNA production and late stages of acute infection and persistent infections with vpiRNAs (Léger et al., 2013). Also during oral infection of *Ae. albopictus* with CHIKV, vpiRNAs became abundant during late stages with characteristic features such as ping-pong signature and the association with predominant hotspots on the genome (Goic et al., 2016). Production of vsiRNAs likely correlates with activation of antiviral defense given the demonstrated antiviral role of the exo-RNAi pathway. On the other hand, in the absence of a clearly demonstrated antiviral role, vpiRNAs may be generated as secondary products when viral replication expands in the infected cells and tissues.

### Expansion of piRNA Genes and Transposon Control

In Aag-2 cells, derived from *Ae. aegypti*, abundant small RNAs were detected that mapped to transposon sequences (Vodovar et al., 2012; Miesen et al., 2015). Most endo-siRNAs were associated with DNA transposons such as miniature inverted terminal elements (MITEs) (Arensburger et al., 2011); piRNAs mainly derived from retrotransposons and showed an antisense bias and ping-pong signature (Vodovar et al., 2012; Miesen et al., 2015). Knock-down experiments established that both *piwi-4* and *piwi-5* were required for production of antisense transposon-derived piRNAs while production of sense piRNAs depended on *ago-3*. Consistent with this, immunoprecipitates of Piwi-5 and Ago-3 were enriched for transposon-derived piRNAs of antisense and sense orientation, respectively. On the other hand, Piwi-4 was relatively depleted for transposon-derived piRNAs which was also observed for vpiRNAs (Miesen et al., 2015). Interestingly, retrotransposons could be classified into different groups based on the dependence of their abundance upon knock-down of particular Piwi-related genes (*piwi-4*, *piwi-5*, *piwi-6*, *ago-3*) and on their association with particular Piwi proteins (Piwi-5, Piwi-6, Ago-3) in immunoprecipitates. Since the genome of *Ae. aegypti* is very rich in transposon sequences (Nene et al., 2007; Arensburger et al., 2011), it can be speculated that the diversification of its piRNA pathway occurred from the need to control parasitic RNAs from different origins. During this process, Piwi-5 and Ago-3 became the main factors for the production of vpiRNAs during viral infections even if the role in antiviral defense remains unclear. The great majority of vpiRNAs during occurring infections are of sense orientation and therefore must be derived from viral genomes of positive sense RNA viruses and viral mRNAs but it is currently unknown how these viral precursors are differentiated from abundant cellular mRNAs and can act as sources for piRNAs (Miesen et al., 2015).

However, despite the abundance of transposon sequences in its genome, only 19% of piRNAs map to transposon sequences in *Ae. aegypti*, in contrast to *Drosophila*, where the proportion is much higher (51%) (Arensburger et al., 2011). Recently, a broader role for piRNAs and PIWI class proteins in the regulation of cellular gene expression in both soma and germline became more apparent in different organisms (Robine et al., 2009; Ishizu et al., 2015; Lewis et al., 2018). In Aag-2 cells, a significant proportion of piRNAs are produced from transcripts of protein-coding genes according to a similar mechanism as for the production of vpiRNAs during arbovirus infection (Girardi et al., 2017). As is the case for transposons (Miesen et al., 2016a), cellular transcripts can be grouped in different classes with respect to production of vpiRNAs, i.e., according to the dependence/association with particular Piwi-related genes/proteins and *ago-3*/Ago-3. Differentiation of the piRNA pathway in *Ae. aegypti* therefore may not only reflect strategies of transposon control but also modes of regulation of gene expression by piRNAs. Interestingly, protein-coding genes can produce piRNAs via a ping-pong mechanism that involves *piwi-5* and *ago-3* as is observed for the production of vpiRNAs (Girardi et al., 2017). It can therefore be asked whether viral (anti)genome strands or mRNAs are recruited to this mechanism by chance or whether it could reflect a still unknown regulatory mechanism.

## Amplification of the Antiviral Rnai Response and Potential Establishment of Immune Memory

### Arboviral DNA Forms

During arboviral (CHIKV, DENV, WNV, LACV) infections of mosquitoes and/or mosquito cell lines, viral DNA forms (vDNAs) are produced by cellular retrotransposon-derived reverse transcriptase (RT) activity (Goic et al., 2016; Nag et al., 2016; Rückert et al., 2019). Synthesis of vDNA was observed to stimulate vsiRNA production especially during the early stages of oral infection of *Ae. albopictus* mosquitoes by CHIKV. While inhibition of RT activity increased mortality in infected mosquitoes, viral replication levels remained unaffected leading to the proposal that production of vDNA forms increased the tolerance (but not the resistance) of mosquitoes to CHIKV infections (Goic et al., 2016). During infection of *Ae. aegypti* mosquitoes with CHIKV, both linear and circular vDNA forms are produced (Poirier et al., 2018). In extension to studies with *Drosophila*, procedures that increase the production of vDNA forms, such as the increased presence of defective viral genomes (DVGs) in the inoculum, could decrease the infection rate of CHIKV during blood feeding of *Ae. aegypti* mosquitoes (Poirier et al., 2018).

Experiments with mosquito cell lines (Aag, C6/36 and *Culex*-derived cell lines) showed that production of vDNA forms did not involve the entire viral genome but rather discrete patches that indicate separate events of template switching of RT activity between template retrotransposon RNA and nearby arboviral genomic RNA or mRNA (Nag et al., 2016; Rückert et al., 2019). Production of vDNA was dependent on the abundance of arboviral genomes and mRNAs since it was found to be enhanced in C6/36 cells where viral titers are typically much higher because of a defect in the exo-RNAi pathway (Morazzani et al., 2012).

### Arboviral Sequences Integrated in Mosquito Genomes

While synthesis of vDNA forms may play a role in antiviral defense during occurring viral infections, sequencing of the genomes of several mosquito species has also established that sequences of RNA viruses can become integrated in the host genomes and possibly maintained as reservoirs of acquired immune memory (Katzourakis and Gifford, 2010; Arensburger et al., 2011; Girardi et al., 2017). Inserted viral sequences are generally known as “endogenous viral elements” (EVEs) but of interest here are “non-retroviral integrated RNA virus sequences” (NIRVS) that correspond to insertions of RNA viruses that lack an RT gene and that are thought to represent very rare events (Ballinger et al., 2014). NIRVS are sparse in the genomes of *Cx. quinquefasciatus* and anopheline species but 10-fold more frequent in *Ae. aegypti* and *Ae. albopictus*; their abundance therefore does not seem to correlate with viral exposure (high in both *Culex* and both *Aedes* species) but may be the result of a combination of the expansion of the piRNA pathway (in *Culex* and both *Aedes* species but not in *Anopheles*) and the load of retrotransposons in the genome (high in both *Aedes* species; intermediate in *Culex*; low in *Anopheles*) (Chen et al., 2015; Palatini et al., 2017). Infection intensity may be an important factor since it would stimulate the formation of vDNA (Olson and Bonizzoni, 2017).

Mechanisms proposed for integration include hitchhiking on retrotransposable elements and via double-stranded break repair (Liu et al., 2010; Fort et al., 2012). A close association of NIRVS and transposable elements is observed in *Aedes* mosquitoes, particularly with long terminal repeat (LTR) retrotransposons of the Gypsy and Pao Bell families (Palatini et al., 2017). The most abundant NIRVS derive from the Flavivirus genus and *Rhabdoviridae* family (Crochu et al., 2004; Fort et al., 2012; Pischedda et al., 2019) with Bunyavirales- and *Reoviridae*-like sequences much more rare (and alphavirus-derived sequences absent; Palatini et al., 2017). Most NIRVS correspond to mosquito-specific viruses that are related to arboviruses (i.e., they do not cycle with vertebrate hosts) but it should be considered that mosquito-specific viruses are more likely to be transmitted vertically through the germline and that only NIRVS integrated in the germline will be passed to the next generation. The occurrence of arboviral NIRVS in genomes of somatic cells would not be passed to the next generation and more difficult to detect.

Interestingly, NIRVS are located in regions of the *Aedes* genome that preferentially produce piRNAs (Arensburger et al., 2011; Girardi et al., 2017; Palatini et al., 2017) and recently were more precisely designated in Aag-2 cells to loci that resemble piRNA clusters from *Drosophila* (Whitfield et al., 2017). NIRVS-specific piRNAs were biased for U at the first position and preferentially of antisense orientation, indicating the potential to interfere with invading viral genomes or mRNAs; for NIRVS derived from rhabdoviruses, a ping-pong signature was also observed. Knock-down and immunoprecipitation experiments in Aag-2 cells established dependence for their formation on *piwi-4*, *piwi-5*, and *piwi-6* and their association with Piwi-5 and Piwi-6 proteins (Palatini et al., 2017). A direct interaction between Phasi Charoen-like virus (PCLV), a bunyavirus that persistently infects Aag-2 cells, and NIRVS-derived (antisense) piRNAs from a piRNA cluster was also demonstrated. VpiRNAs of sense orientation are produced from different regions of the PCLV genome but, in a region of common sequences between NIRVS and the virus, a peak of sense piRNA production from the virus was separated by 10 bp from a peak of antisense piRNAs apparently derived from the NIRVS, an interaction which is consistent with the ping-pong mechanism (Whitfield et al., 2017). This observation indicates the possibility that NIRVS-derived piRNAs contribute to the regulation of the persistent infection of PCLV in Aag-2 cells.

Besides piRNA clusters, NIRVS can also be mapped to coding sequences of genes and be expressed, with a possible role in antiviral immune function (Pischedda et al., 2019). Examples of such genes are annotated as RdRp- and nucleocapsid-encoding genes of rhabdoviruses.

## Systemic Response Triggered by Dicer-2

Besides its crucial function in the antiviral exo-RNAi pathway Dicer-2 was also proposed to have a role as a PAMP-recognition receptor (PRR) during viral infections. The DExD/H helicase domains of Dcr-2 enzymes in insects and the RIG-I-like receptors (RLRs), that trigger interferon I responses in mammals, were shown to be phylogenetically related and could be grouped together in the family of duplex RNA-activated ATPases (DRAs) family (Luo et al., 2013). A recent study mainly focusing on *Drosophila* suggests that Dcr-2 may preferentially interact with DVGs in the cytoplasm and promote the interaction with retrotransposons to synthesize vDNA (Poirier et al., 2018). Evidence also exists that vDNA could act as a systemic signal in mosquitoes and prime the immune response against infection of homologous virus (Goic et al., 2016; Poirier et al., 2018).

In addition, an involvement of the helicase domain of Dcr-2 was shown in the production of Vago, a secreted peptide with antiviral function characterized by a single von Willebrand C domain. It was observed that infection of WNV in a cell line of *Cx. quinquefasciatus* (Hsu) induced the expression of the secreted peptide CxVago in a Dcr-2-dependent manner (Paradkar et al., 2012). The induction of CxVago expression was also observed after injection of *Cx. pipiens* f. *molestus* mosquitoes with Kunjin virus (KUNV; *Flaviviridae*) and after infection of the *Ae. albopictus* RML12 cell line with DENV (Paradkar et al., 2012, 2014), but not after injection of *Ae. aegypti* mosquitoes with DENV (Asad et al., 2018).

In the Hsu cell line, it was demonstrated that CxVago exhibited antiviral effects through the induction of the JAK/STAT pathway (Paradkar et al., 2012). In order to induce the STAT-dependent target gene *vir-1* and to inhibit WNV replication, CxVago signaling required CxJAK (Hopscotch homolog) but not the classical CxDome receptor for the unpaired ligands, leading to the speculation of the antiviral response occurring through an alternative JAK receptor (Paradkar et al., 2012). Another study employing the Hsu cell line documented that activation of CxVago production following WNV infection required the TRAF adaptor protein and the Rel2 NF-κB transcription factor and confirmed the involvement of Dcr-2 in the activation mechanism (Paradkar et al., 2014). In another cell line, Aag-2, the antiviral effect of *Wolbachia* infection was shown to be mediated by up-regulation of *AeVago1* (Asad et al., 2018).

The mechanistic details of the induction of CxVago by arbovirus infection and its activation of the JAK/STAT signaling pathway and antiviral effects have only been demonstrated in the Hsu cell line and not in mosquitoes. Similarly, while *AeVago1* is induced during *Wolbachia* infection in *Ae. aegypti* mosquitoes, its functional involvement in repression of DENV infection remains to be demonstrated *in vivo*.

Other mechanisms of spread of an antiviral signal have been proposed, for instance in the U4.4 cell line, that requires direct contact between adjacent cells. The systemic signal, that is proposed to move from cell to cell through gap junctions or cytoplasmic bridges, may consist of viral dsRNAs/siRNAs. During infections with recombinant SFV expressing tombovirus p19 (which sequesters siRNA but not dsRNA) spread of infection was stimulated rather than viral replication in initially infected cells (Attarzadeh-Yazdi et al., 2009).

## The Function of the miRNA Pathway During Arbovirus Infection

Comparison of the rates of evolution among *Drosophila* species showed the accelerated evolution of genes in the exo-RNAi pathway with respect to miRNA genes, which was contributed to the molecular arms race between host antiviral response and pathogenic virus infection (Obbard et al., 2006, 2011). By contrast, another study demonstrated that genes in both miRNA and exo-RNAi pathways underwent rapid diversifying selection among different populations of *Ae. aegypti* (Bernhardt et al., 2012). While the causes of such accelerated evolution of miRNA genes remain to be established and may not be related to arbovirus infections, it is nevertheless of interest to consider studies that investigated the involvement of miRNAs during arbovirus infections (reviews by Asgari, 2014; Hussain et al., 2016; Monsanto-Hearne and Johnson, 2018). Intriguingly, a significant positive correlation was found between the midgut escape barrier for DENV infection and nucleotide diversity indices in *dcr-2* but also in *dcr-1* (Bernhardt et al., 2012).

Many studies concerning the interaction of arbovirus infection with the miRNA pathway are descriptive and involve the cataloging of miRNAs that show differential expression following arbovirus infection in mosquitoes (mostly whole body but also midgut and saliva; Zhou et al., 2014; Maharaj et al., 2015; Liu et al., 2016) or mosquito-derived cell lines, which is followed by the prediction of cellular target genes by *in silico* analysis or by correlation of expression with host transcripts (review by Monsanto-Hearne and Johnson, 2018). A complex response is often recorded in which particular patterns can be observed during the course of infection with different arboviruses (DENV, CHIKV, ZIKV) (Saldaña et al., 2017; review by Monsanto-Hearne and Johnson, 2018) but for which the functional relevance *in vivo* remains untested. Gene ontology (GO) and Kyoto Encyclopedia of Genes and Genomes (KEGG) analysis reveals the identification of immune genes among different predicted cellular targets (Liu et al., 2015, 2016; Xing et al., 2016). However, in some studies, no significant changes in miRNA abundance are observed following arbovirus infection (Miesen et al., 2016b; Ferreira et al., 2018).

Functional studies of the interaction of miRNAs with arbovirus infection were carried out in Aag-2 cells that are amenable to RNAi-mediated gene silencing and can be easily transfected with reporter/sensor/expression constructs. Such studies established a miRNA-mediated mechanism by which *Wolbachia* bacterial endosymbionts may inhibit DENV infection in *Ae. aegypti* mosquitoes, i.e., through the induction of aae-miR-2940 and inhibition of its target *Dnmt2* (encoding a DNA methyltransferase) (Zhang et al., 2013). In another study, infection of mosquito cell lines by the Kunjin strain of WNV becomes restricted following down-regulation of aae-miR-2940 and its target gene encoding the metalloprotease m41 FtsH (which is positively regulated by the miRNA) (Slonchak et al., 2014). Also aae-miR-375 enhances DENV infection in Aag-2 cells, an observation which can be correlated with increased expression of *cactus*, which encodes an inhibitor of the immune regulator REL1, an NF-κB transcription factor (Hussain et al., 2013). Regarding the mosquito-borne alphavirus North American eastern equine encephalitis virus (EEEV), it was reported that the integrity of a region in the 3′UTR, which coincides with binding sites for miR-142-3p in mammalian cells, was important for viral replication in mosquito C6/36 cells and infection of *Ochlerotatus taeniorhynchus* vector mosquitoes (Trobaugh et al., 2014).

Functional studies in *Ae. albopictus* mosquitoes through the injection of miRNA mimics or antagomirs revealed a positive role for the midgut-specific aae-miR-281 to regulate DENV replication (Zhou et al., 2014). Sensor constructs that harbor aae-miR-281 target sites from the 5′-UTR of DENV were positively affected following administration of aae-miR-281 in C6/36 cells, indicating a direct interaction between miR-281 and the DENV genome (Zhou et al., 2014). Finally, all functional studies employing flavivirus should take into account that sfRNA could also act as an inhibitor of the miRNA pathway, in addition to the exo-RNAi pathway, as demonstrated for WNV-sfRNA in *Drosophila* S2 cells (Schnettler et al., 2012) (see also section “SfRNA” for discussion of the possible function of sfRNA as VSR).

miRNAs can also be produced by viruses although it is considered rather unlikely that RNA viruses produce miRNAs (discussion by Umbach and Cullen, 2009). During infection of Aag-2 and C6/36 cells with the Kunjin strain of WNV, a viral miRNA (KUN-miR-1) is produced that is derived from the 3′ stem-loop located at the very end of the 3′-UTR or sfRNA (Hussain et al., 2012). KUN-miR-1 was proposed to stimulate Kunjin virus infection through positive regulation of the target gene GATA-4, which is a transcriptional regulator of genes involved in lipid metabolism. Another example is DENV-vsRNA-5 that corresponds to the first stem-loop structure at the beginning of the 3′-UTR of sfRNA of DENV (Hussain and Asgari, 2014). DENV-vsRNA-5 is proposed to have an autoregulatory function as it targets the ORF of DENV NS1. However, the latter work was criticized since DENV-vsRNA-5 was expressed at too low levels to be able to act as a stoichiometric inhibitor like other miRNAs (Skalsky et al., 2014). Another study has predicted several potential viral miRNAs from DENV *in silico* (Ospina-Bedoya et al., 2014) but they could not be experimentally verified (Miesen et al., 2016a). Production of miRNAs by arboviruses remains therefore a contentious issue and so far functional data were obtained in cell lines but not in mosquitoes.

## RNAi and the Antiviral Defense Against Mosquito-Specific Viruses

With the advent of deep sequencing techniques and an increasing interest in the mosquito microbiome, a considerable number of new viruses were identified that are related to known arboviruses belonging to the genera Flavivirus and Alphavirus, the family *Rhabdoviridae* and the order Bunyavirales (Blitvich and Firth, 2015; Bolling et al., 2015; Longdon et al., 2015; Marklewitz et al., 2015; Halbach et al., 2017). In general, it was observed that the taxonomic groups that encompass known arboviruses contain additional viruses that can be considered either as mosquito-specific viruses (i.e., restricted to only mosquitoes and no transmission to vertebrates) or as having “no known vector” (i.e., likely restricted to only vertebrates). In addition, many new viruses that infect mosquitoes were isolated that have a taxonomy not related to any current known arboviruses (Ma et al., 2011; Zirkel et al., 2013; Vasilakis et al., 2013; Parry and Asgari, 2018).

Because such mosquito-specific viruses do not require a period of persistence to acquire competence for transmission to a vertebrate host, they can be expected to cause pathogenic infections in mosquitoes. However, in practice, no such pathogenic infections were manifest in most instances since it was found that vertical (transovarial) transmission was the most frequent pathway for viral dispersion in the mosquito population (Blitvich and Firth, 2015; Longdon et al., 2015; Halbach et al., 2017; Parry and Asgari, 2018). Thus, mosquito-specific viruses may not differ much from arboviruses since a long-term state of equilibrium between virus and host is desirable in both cases, although differences in tissue tropism (salivary glands versus gonads) may be apparent. In such case, the same principles with respect to avoidance of clearance by the RNAi mechanism or recognition by the immune response will be found during both arbovirus and persistent mosquito-specific virus infections.

Nevertheless, it was found that some mosquito-specific viruses can encode inhibitors of RNAi, indicating their potential to spread by horizontal transmission and to cause pathogenic infections [e.g., mosinovirus (MoNV, *Nodaviridae*; Schuster et al., 2014] and *Culex* Y virus (CYV, Birnaviridae; van Cleef et al., 2014; Franzke et al., 2018). For other viruses, the presence of a VSR gene could be inferred after analysis of the profile of viral small RNAs (Aguiar et al., 2015).

During comparisons between infections of DENV and the mosquito-specific flavivirus CFAV in the mosquito cell lines C6/36 and Aag-2, much higher levels of viral small RNAs were detected for CFAV which reflected its higher replication levels (Scott et al., 2010). As is the case for DENV infections, both vsiRNAs and vpiRNAs were observed in CFAV infections of which the latter showed a ping-pong signature (A10 but not U1) in Dcr-2-defective C6/36 cells. During CFAV infections much more prominent hot spots of viral small RNA production were observed as during DENV infections.

In *Anopheles* mosquitoes, production of vsiRNAs is the predominant pathway for some infections of mosquito-specific flaviviruses, while in other infections viral small RNAs of many different sizes are produced corresponding to the positive strand and with a minor peak at 21 nt (Colmant et al., 2017). During infections of *Ae. aegypti* cells with the negative-strand *Aedes* anphevirus (AeAV; unclassified in Mononegavirales order), the production of vsiRNAs is dwarfed by the presence of vpiRNAs that show a clear ping-pong pattern (A10 genome, U1 antigenome) (Parry and Asgari, 2018). As also observed in arboviruses with negative-strand genomes (Léger et al., 2013), hot spots for abundant vpiRNA production can be observed.

In *Culex-*derived cell lines, different patterns of viral small RNA production are observed following infection with the mosquito-specific flavivirus Calbertado virus (CLBOV; only vsiRNAs) and the mosquito-specific rhabdovirus Meridavirus (MERDV; both vsiRNAs and vpiRNAs, the latter with positive strand-bias and ping-pong signature) (Rückert et al., 2019). These differences in viral small RNAs are considered to be caused by infections with viruses of different families (*Flaviviridae* and *Rhabdoviridae*) while it is expected that infections with arboviruses and mosquito-specific viruses of the same family will generate similar patterns of viral small RNAs in similar hosts. Mosquito-specific (CLBOV) and arboviral (WNV) flaviviral infection of *Culex tarsalis*-derived CT cells indeed results in the generation of very comparable profiles of viral small RNAs (Rückert et al., 2019).

The high similarity between infections of mosquito-specific viruses and arboviruses that belong to the same virus family is also observed with respect to their sensitivity to *Wolbachia* co-infection. While both mosquito-specific and arboviral flavivirus (with (+) ssRNA genome) infections are effectively cleared by *Wolbachia*, bunyaviruses [with segmented (-) or ambisense ssRNA genome] are resistant to the endosymbiont and the resistant phenotype applies to both mosquito-specific and arboviral species (Schnettler et al., 2016).

In contrast to the significant production of both vsiRNAs and candidate piRNAs (the latter albeit without ping-pong signature), it was reported that miRNA expression was very little affected during infection of *Ae. aegypti* with the mosquito-specific flavivirus Palm Creek virus (PCV) (Lee et al., 2017). Furthermore, inhibition of the few miRNAs that showed differential expression did not result in any effects on PCV replication. As is the case for arboviruses, this study suggests at best a minor role for miRNAs in the regulation of mosquito-specific virus infection (see also section “The Function of the miRNA Pathway During Arbovirus Infection”).

The above observations of viral small RNA accumulation indicate that mosquito-specific viruses can activate both the exo-RNAi (siRNA) and piRNA mechanism while other unknown RNA degradation pathways are also revealed. In conclusion, there is no evidence for significant differences between infections of arboviruses and mosquito-specific viruses belonging to the same virus family with respect to the RNAi response and other resistance mechanisms, although higher amounts of viral small RNAs may accumulate in infections with mosquito-specific viruses because of their higher replication level, potentially leading to pathogenicity.

## Engineering the RdRP Engines of Arbovirus Replication

The ability of (arbo)viruses to induce RNAi effects and achieve VIGS is mainly determined by two properties, i.e., the encoding of VSR genes and the characteristics of the RdRp enzymes with respect to kinetics and replication capacity (O’Neal et al., 2014). Both properties should be coordinated such that an optimal balance is achieved between viral replication and avoidance of the immune response to promote viral persistence (Randall and Griffin, 2017). While the importance of VSRs to regulate persistence and pathogenicity was already mentioned (section “Evidence for Presence of VSR Genes in Arboviral Genomes”), this part will focus on the effect of the properties of the RdRP enzyme on RNA virus infections.

RdRP enzymes of RNA viruses have high error rates which result in mutation frequencies of 10^-4^ mutations per nucleotide copied (Sanjuan et al., 2010). Error rates are influenced by environmental factors and can be stimulated by nucleoside analogs (te Velthuis, 2014). Few data exist with respect to the characteristics of RdRP enzymes of arboviruses but recently several studies were published that investigate the effects of lower or higher fidelity RdRp variants on infection properties of alphaviruses such as SINV and CHIKV (Coffey et al., 2011; Rozen-Gagnon et al., 2014; Stapleford et al., 2015; Poirier et al., 2016).

Research focused on the role of a residue in the palm domain of RdRp of alphaviruses that regulates its replication fidelity (Rozen-Gagnon et al., 2014; Stapleford et al., 2015). When tested in *Ae. aegypti* mosquitoes, an antimutator variant (exhibiting higher fidelity) of CHIKV exhibited lower infection and dissemination titers which was attributed to reduced genetic diversity (Coffey et al., 2011). On the other hand, mutator variants (with lower fidelity) of CHIKV and SINV displayed replication defects in mosquito cells and reverted to wild-type or other replication competent variants (Rozen-Gagnon et al., 2014). During infection of *Drosophila* S2 cells with low fidelity SINV, by contrast, no replication defect or reversion to wild-type was observed although mutator variants presented significantly lower titers than wild-type. These data illustrate the potential for engineering RdRp enzymes to affect infectivity and replication and also indicate the existence of cell-specific effects.

The (negative) effects of both antimutator and mutator variants were initially thought to be mainly caused by the reduction and augmentation of genetic diversity leading to the proposal that RdRp enzymes of RNA viruses have become optimized during evolution to be neither too accurate nor too erroneous. However, in mammalian cells it was also demonstrated that mutator variants of SINV show an increased recombination rate leading to defective interfering (DI) particle production (Poirier et al., 2016). Whether this phenomenon also occurs in mosquito cells and how it can impact the RNAi response and the establishment of persistent infections remains to be investigated.

## Conclusion

Arbovirus infections of mosquitoes are characterized by the absence of pathogenic effects that allow their spread within the mosquito body to the salivary glands from where infection of new vertebrate hosts can be initiated. Despite the persistent character of the infections, functional viral small RNAs were produced by recombinant viruses that could confer resistance to secondary infections by unrelated arboviruses (Gaines et al., 1996; Olson et al., 1996, 2002; Powers et al., 1996; Adelman et al., 2001). However, it was not determined whether recombinant arboviruses could induce silencing of cellular genes in mosquitoes or mosquito cell lines.

Because of the persistent character of their infections, engineered arboviruses or related mosquito-specific viruses could be developed as effective gene silencing vectors in mosquitoes. RNAi is a very powerful tool to carry out reverse genetics studies but in many cases the delivery of the dsRNA trigger is limiting (Scott et al., 2013; Whitten and Dyson, 2017). Engineered RNA viruses that have incorporated cellular gene fragments could be developed as efficient silencing vectors for analysis of gene function on a gene-by-gene basis. Moreover, if high efficiency of oral infection can be achieved, libraries of viral silencing vectors can be applied in large-scale screening experiments for interrogation of gene function in physiological and developmental processes. In such applications, however, it is extremely important to avoid non-specific effects caused by damage of viral replication and activation of innate immunity, which is the reason why persistent infections of arboviruses can serve as an excellent model for the development of viral silencing vectors. In a second type of application, viral silencing vectors that target essential genes of the host can be employed as novel types of insecticides since incorporation of fragments of essential host genes in the viral genomes is predicted to increase the virulence and to induce lethality (Taning et al., 2018). Because of the specificity of hybridization of the vsiRNAs to the targeted mRNAs, knock-down of essential genes and associated lethal effects will be observed in only single or closely related species, which is an increased safety feature. However, the application of engineered viruses as specific insecticides needs careful evaluation with respect to interaction with non-target species, the stability of the genetic material and possible recombination with co-infecting natural viruses (Kolliopoulou et al., 2017).

Understanding of the parameters that determine the persistent character of arbovirus infections may have important implications for the design of silencing vectors in insects in general. In this review, an extensive discussion is presented regarding the interaction of arbovirus infections with the mosquito RNAi machinery that can serve as a background for the improvement of the capacity of arbovirus vectors to induce specific gene silencing. As already mentioned in other studies (O’Neal et al., 2014; Kolliopoulou et al., 2017), two important factors have emerged that determine the capacity of viruses to function as gene silencing factors: (1) the presence of VSR genes in the virus genome, and (2) the replication capacity of the virus mainly determined by the properties of the RdRp.

It remains under debate whether arboviruses encode canonical VSR genes. However, other strategies for evading the RNAi response can exist such as the shielding of viral replication complexes in membrane structures (Den Boon and Ahlquist, 2010) or the activation of other immune pathways such as JAK-STAT and Imd (Waldock et al., 2012). On the other hand, when arbovirus genomes are engineered to contain a well-characterized VSR gene from an unrelated RNA virus, infections become much more virulent due to higher viral replication (Myles et al., 2008). These experiments indicate that it is possible to modulate the RNAi response in arbovirus vectors through the artificial introduction of VSR genes of different strengths (O’Neal et al., 2014). It remains to be determined whether the gene silencing activity of arboviruses can be improved by the incorporation of VSR genes. An optimal level of inhibition of RNAi may exist that achieves more efficient silencing than that observed for wild-type arboviruses because of the higher levels of viral replication that are achieved. However, the occurrence of cellular damage and the activation of innate immune pathways need to be avoided because they will confuse the interpretation of the phenotypes caused by specific gene silencing.

Besides activation of the exo-RNAi pathway, which is thought to carry out the gene silencing effects, arbovirus infections in mosquitoes also can result in the abundant production of vpiRNAs. Since their involvement in antiviral defense was not clearly demonstrated (Varjak et al., 2018b), the function of vpiRNAs during viral infection remains to be elucidated. Their production may be a secondary effect of the expansion of PIWI genes in aedine and culicine mosquitoes since canonical vpiRNAs (with ping-pong signature) do not appear during arbovirus infections of anopheline mosquitoes, sandflies or midges that (presumably) have a more standard set of PIWI genes (Myles et al., 2009; Schnettler et al., 2013b; Ferreira et al., 2018; Dietrich et al., 2017b). The production of vpiRNAs in aedine and culicine mosquitoes occurs with specific patterns according to the type of virus (alphavirus, flavivirus, or bunyavirus) (Varjak et al., 2018b). While studies of the function of piRNAs have mainly focused on their role in transposon silencing in the germline, the production of piRNAs in somatic tissues of many insects was revealed recently (Lewis et al., 2018). piRNAs in somatic tissues do not only correspond to transposable elements but also map to protein-coding genes that suggest unknown functions in the regulation of gene expression. Open questions include whether canonical vpiRNAs are also produced during viral infections in insects that do not have an expanded PIWI gene set and whether they may function in the regulation of viral and cellular gene expression. The investigation of the functions of vpiRNAs, however, may be aggravated by their overlap in sequence with vsiRNAs. A major argument against the antiviral function of the piRNA pathway is that VSRs targeting this pathway were never identified.

An interesting observation is the production of viral DNA forms during infection which is thought to be related to antiviral defense (Nag et al., 2016; Poirier et al., 2018). This phenomenon was reported in *Drosophila* and mosquitoes and may be related to the abundance of retrotransposons encoding active RT enzymes in the insect genomes (Palatini et al., 2017). Viral DNA forms may be intermediates in the process of integration of viral sequences in insect genomes where they may constitute some type of immune memory against viral infections, possibly through the production of piRNAs (Olson and Bonizzoni, 2017). Integrated viral sequences that were found generally do not represent current infections and evidence of an antiviral function of EVEs and NIRVs to modulate recent arboviral infections is lacking (Varjak et al., 2018b).

Besides its role in the exo-RNAi pathway, Dcr-2 was also found to have a function in the systemic antiviral response. The helicase domain of Dcr-2 functions as a dsRNA sensor and initiates a signaling cascade for the production of secreted antiviral signaling peptides (Paradkar et al., 2012, 2014), similar to cytosolic dsRNA sensors in mammals (Luo et al., 2013). While this review focused on the antiviral RNAi pathway, abundant evidence exists regarding the existence of several other antiviral pathways in mosquitoes (e.g., Xi et al., 2008; Souza-Neto et al., 2009; Rodriguez-Andres et al., 2012; Blair and Olson, 2014; Cheng et al., 2016; Clem, 2016) that are beyond the scope of this review. Of relevance is the observation that in insects, in contrast to vertebrates, very few PRRs were identified that directly interact with PAMPs produced during viral infections (e.g., Deddouche et al., 2008; Nakamoto et al., 2012). Instead, general damage that occurs during excessive viral replication and virion production may induce a stress response that will curtail the spread of viral infections (Moreno-García et al., 2014). From this viewpoint, control of the activity of RdRp enzymes of RNA viruses is essential for evasion of innate immunity pathways. RdRp enzymes of alphaviruses that differ from wild-type with respect to fidelity were described (Rozen-Gagnon et al., 2014) but how these and other types of mutants may interact differentially with the RNAi machinery and other innate immune pathways need to be investigated in more detail.

While arboviruses establish persistent infections and trigger an RNAi response in mosquitoes, their development as silencing vectors may not be practical because of their capacity to cause disease in humans and livestock. Fortunately, mosquito-specific viruses related to arboviruses were described for which reverse genetics systems are available (Nasar et al., 2015; Junglen et al., 2017). In addition, the technique of circular polymerase extension cloning allows the straightforward assembly of reverse genetics systems for RNA viruses without the need for molecular cloning using bacterial strains (Quan and Tian, 2009; Piyasena et al., 2017). Since mosquito-specific viruses generally also cause persistent infections in mosquitoes that resemble persistent infections by arboviruses (albeit with different tissue tropism), they represent a more safe alternative to arboviruses. However, their replication properties, interaction with innate immunity and RNAi machinery, and host range need to be investigated to a much greater extent.

## Author Contributions

JL and LS conceived the idea, and designed and wrote the manuscript. AK designed the figure and contributed to the revision of the text. GS revised and contributed to improve the final version of the manuscript. All authors read and approved the final version of the manuscript.

## Conflict of Interest Statement

The authors declare that the research was conducted in the absence of any commercial or financial relationships that could be construed as a potential conflict of interest.
